# A new synthesis of indolo[2,3-*b*]quinolines from 3-acetyl-*N*-alkyl-2-chloroindoles with 2-aminobenzophenone†

**DOI:** 10.1039/d4ra05176a

**Published:** 2024-09-26

**Authors:** Hong Zhang, Yunhe Jiang, Xiaoxue Sun, Tianyu Liang, Xiang Wang, Yang Li

**Affiliations:** a Experiment & Equipment Administration Center, Bohai University Jinzhou 121000 P. R. China; b College of Chemistry and Materials Engineering, Bohai University Jinzhou P. R. China bhuzh@163.com; c Key Laboratory of Biotechnology and Bioresources Utilization of Ministry of Education, Dalian Minzu University Dalian 116600 P. R. China

## Abstract

A new synthesis of *N*-alkyl- and 11-phenyl-modified indolo[2,3-*b*]quinolines was achieved *via* PEG-400-promoted and visible light-induced one-step reaction of 3-acetyl-*N*-alkyl-2-chloroindoles with 2-aminobenzophenone in 40% methanol aqueous solution. The merits of the protocol include cost efficiency, convenience, and eco- and user-friendliness.

## Abstract 2

A new synthesis of *N*-alkyl- and 11-phenyl-modified indolo[2,3-*b*]quinolines was achieved *via* PEG-400-promoted and visible light-induced one-step reaction of 3-acetyl-*N*-alkyl-2-chloroindoles with 2-aminobenzophenone in 40% methanol aqueous solution.

## Graphical abstract

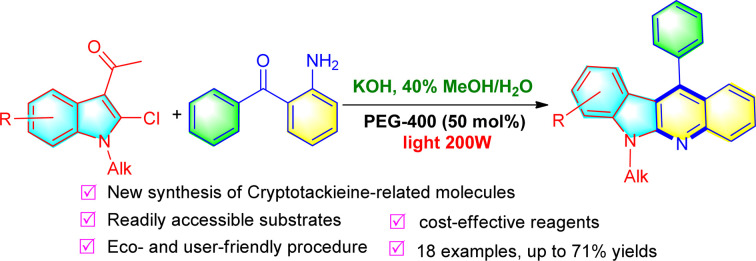

## Introduction

**Fig. 1:**
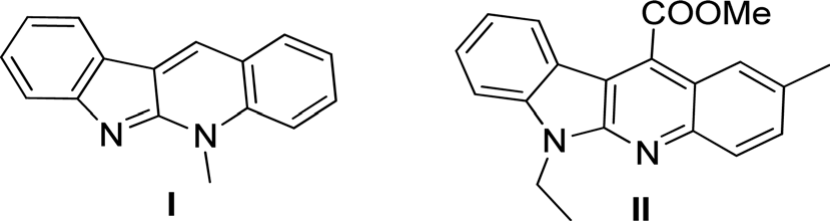
Structures of cryptotackieine (I) and its derivative II.

The tetracyclic-fused indolo[2,3-*b*]quinoline system, as the core structure of the natural alkaloid cryptotackieine (5-methyl-5*H*-indolo[2,3-*b*]quinoline) (I, Fig. 1) isolated from West African plant Cryptolepis sanguinolenta^1,2^ has been renowned for its powerful DNA intercalation capability and topoisomerase inhibition property,^3^ thereby exhibiting potent antiplasmodial activity.^4^

In comparison with cryptotackieine, despite having not been found in nature, some synthesized *N*-alkyl-substituted indolo[2,3-*b*]quinoline derivatives have been discovered to explicitly display significant biological activities such as antimicrobial, cytotoxic activities *in vitro* and antitumor properties *in vivo*.^5–7^ For example, Sunke *et al.* reported that the synthesized methyl 2-(6-ethyl-2-methyl-6*H*-indolo[2,3-*b*]quinolin-11-yl)acetate (II, Fig. 1) showed the most promising cytotoxicity against cancer cells and apoptosis inducing properties.^8^ Furthermore, the scaffold has been frequently taken part in the design and development of modern pharmaceuticals and pesticides.^9^

In light of the unique structural array and the huge pharmaceutical applications, during the past decades it has motivated a surge of synthetic interest in developing novel and facile pathways to the synthesis of the intriguing types of indolo[2,3-*b*]quinoline derivatives to expand the molecular library of this tetracyclic molecular template for the potential application in the current drug discovery programs. Generally, there are three distinct strategies for the construction of the basic indolo[2,3-*b*]quinoline skeleton, including (1) building up the quinoline moiety starting from an indole-based partner, which is also the most common and widely reported scenario;^10–23^ (2) constructing the indole moiety by using a quinoline-based substrate as the coupling partner;^24–27^ and (3) fabricating the indolo[2,3-*b*]quinoline skeleton through annulation reaction using non-quinoline and non-indole substrates.^28–31^

Despite the significant progress and fruitful access to indolo[2,3-*b*]quinolines, these existing approaches apparently suffered either one or more of the disadvantages such as toxic & costly metal catalysts, multi-step synthetic pathway, harsh and stringent reaction conditions, poor atom economy and sustainability, difficultly available and highly functionalized reactants, tedious product isolation procedures and low product yields, which have limited their practical applications. For these reasons, the development of more efficient, eco-friendly and convenient strategies for the construction of these useful scaffolds is still highly desirable. Taking these concerns into account, it would be very attractive for the development of a more efficient and sustainable strategy for the synthesis of indolo[2,3-*b*]quinolines. As a consequence, we would like to describe herein an improved synthesis of this type of indolo[2,3-*b*]quinolines by using 40% methanol aqueous solution as reaction medium with KOH as base and PEG-400 as additive under visible light radiation conditions. The fascinating merits of the synthetic approach include cost efficiency, convenience, and eco- and user-friendliness, avoiding the use of toxic, expensive and hazardous catalysts/reagents, thereby enhancing the greenness and practicability of the transformation.

## Results and discussion

**Scheme 1:**
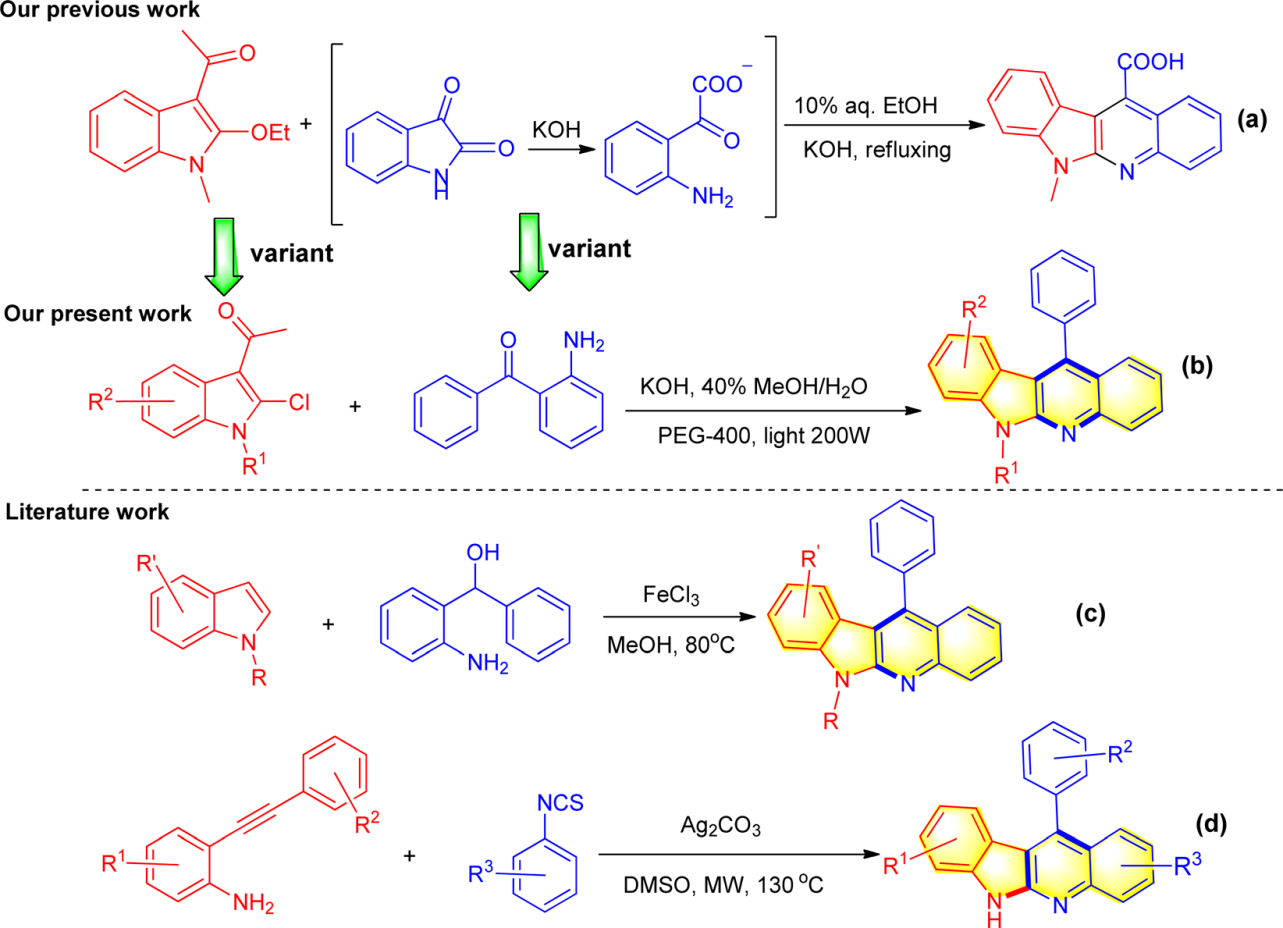
Our work and some related synthetic routes for indolo[2,3-*b*]quinoline derivatives.

In fact, in our previously work we have reported a serendipitous and unexpected reaction between 3-acetyl-2-ethoxyindole with isatins for the green and direct synthesis of indolo[2,3-*b*]quinoline in 10% aqueous ethanol solution with KOH as base as shown in Scheme 1a.^32^

This reaction proceeded through a unique sequence, involving essentially the ring opening of isatins to produce 2-(2-aminophenyl)-2-oxoacetate, followed by cyclization with 3-acetyl-2-ethoxy-*N*-methylindole, wherein the 2-ethoxy substituent acted as a leaving group. Thus, in the context of our continuing interest in the construction of the interesting type of tetracyclic-fused quinolines, we envisioned that the readily available 3-acetyl-2-chloroindole and 2-aminobenzophenone might have such reactivity as well to give rise to the corresponding 11-phenyl-6*H*-indolo[2,3-*b*]quinoline derivatives in a single operation, wherein 3-acetyl-2-chloroindole served as an alternative of 3-acetyl-*N*-methyl-2-ethoxyindole, and 2-aminobenzophenone as a variant of 2-(2-aminophenyl)-2-oxoacetate (Scheme 1b). It is worthy to mention that with respect to this type of derivatives, Yan *et al.* ever reported FeCl_3_-promoted Friedel–Crafts/intramolecular cyclization of aminophenyl alcohols and indoles for the successful synthesis of 11-phenyl-6*H*-indolo[2,3-*b*]quinoline derivatives (Scheme 1c).^33^ Ali *et al.* also described the similar synthesis involving Ag_2_CO_3_ mediated cascade annulation of 2-(phenylethynyl)aniline with phenyl isothiocyanate under microwave heating conditions (Scheme 1d).^34^ Although both methods have been attractive, the required substrates were unstable and less readily accessible, limiting the efficiency and applications.

Our investigation was commenced with the reaction of 3-acetyl-2-chloro-*N*-methylindole (1a) and (2-aminophenyl)(phenyl)methanone (2) as a model to explore the optimized reaction conditions with the results being summarized in Table 1. At the initial stage of this study, we conducted the model reaction in neat water medium with the presence of KOH as the base with the aim of achieving green synthesis. However, no formation of the desired product was observed due to the poor solubility and the starting materials were recovered unchanged (entry 1, Table 1). Further, the addition of some phase-transfer catalysts such as BTEAC or TBAB was also resulted in the failure to obtain the product (entries 2 and 3, Table 1). Thus, in order to improve the solubility and meanwhile retain the simplicity and environmentally benign procedure, the reaction was attempted to be carried out in a methanol–water system with different methanol volume fractions (entries 4–7, Table 1). Interestingly, the use of MeOH aqueous solution as reaction medium indeed promoted the occurrence of the reaction to the utmost extent upon using 40% methanol aqueous solution as the reaction medium. However, the low yield of 31% was still not satisfactory as we expected. This encouraged us to test other organic solvents to find a suitable mixed system for the reaction. Unfortunately, further employing other organic solvents such as ethanol, acetone, 1,4-dioxane and DMF resulted in no further improvement in the product yield. Attempting to use other bases such as NaOH, K_2_CO_3_, Cs_2_CO_3_, and LiOH was frustrated as well by either poor yields or intractable complex mixtures from which it is difficult to separate any desired product in appreciable yield (entries 8–11).

**Table 1:** Exploring the reaction conditions on the synthesis of 3a^a^

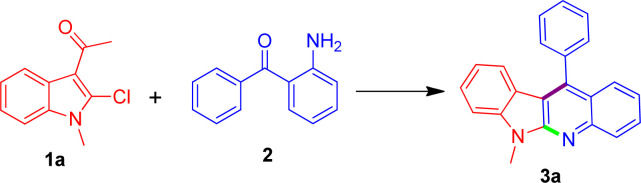					
Entry	Solvent	Base	Additive	Light/W	Yield^b^/%
1	H_2_O	KOH	—	—	N.R.^c^
2	H_2_O	KOH	BTEAC	—	N.R.
3	H_2_O	KOH	TBAB	—	N.R.
4	10% MeOH	KOH	—	—	14
5	20% MeOH	KOH	—	—	21
6	40% MeOH	KOH	—	—	31
7	60% MeOH	KOH	—	—	23
8	40% MeOH	NaOH	—	—	19
9	40% MeOH	K_2_CO_3_	—	—	Trace
10	40% MeOH	Cs_2_CO_3_	—	—	Trace
11	40% MeOH	LiOH	—	—	10
12	40% MeOH	KOH	10 mol% PEG-400	—	31
13	40% MeOH	KOH	20 mol% PEG-400	—	31
14	40% MeOH	KOH	40 mol% PEG-400	—	37
15	40% MeOH	KOH	50 mol% PEG-400	—	41
16	40% MeOH	KOH	60 mol% PEG-400	—	39
17	40% MeOH	KOH	50 mol% PEG-400	60	47
18	40% MeOH	KOH	50 mol% PEG-400	100	52
19	40% MeOH	KOH	50 mol% PEG-400	150	64
**20**	**40% MeOH**	**KOH**	**50 mol% PEG-400**	**200**	**67**
21	40% MeOH	KOH	50 mol% PEG-400	300	61

a Reaction conditions: 1a (0.5 mmol), 2 (0.55 mmol) under refluxing temperature.

b Isolated yields.

c N.R. means no reaction.

After many trials, we found that the addition of PEG-400 as an additive to the reaction mixture appeared to be favorable to push this reaction forward. Through a simple optimization (entries 12–16, Table 1), the best result was obtained when using 50 mol% PEG-400 giving a moderate yield of 41% (entry 15, Table 1). However, no more increase in the yield was observed by further increasing the amount of PEG-400 even to neat PEG-400 or employing other PEGs such as PEG-200, PEG-600 and PEG-800. Consequently, attempts to further improve the product yield were still highly desirable. Recently, there are a few reports involving visible light-induced cyclisation reaction for the synthesis of quinoline derivatives.^35–37^ Accordingly, the versatility of the visible-light-mediated synthesis of quinoline prompted us to explore the feasibility of applying visible-light irradiation in our synthesis. Interestingly, it was found that the application of visible-light irradiation did afford an evident amelioration for the formation of the tetracyclic indolo[2,3-*b*]quinoline skeleton, in which the highest yield of 67% was obtained when using 200 Watt visible light radiation after evaluating the effect of different visible light powers on the product yield (entries 17–21, Table 1). In addition, we also carried out the model reaction under the individual 200 Watt visible light radiation with the absence of PEG-400, whereas in this case a reduced yield was obtained. Thus, the standardized conditions of 40% methanol aqueous solution with the presence of KOH as base and 50 mol% PEG-400 as additive under 200 Watt visible light radiation were regarded as the most appropriate reaction conditions for the model reaction.

Thereafter, due to the good yield obtained under the above optimal reaction conditions, we decided to extend the substrate scope to some other substituted 3-acetyl-*N*-alkyl-2-chloroindoles to demonstrate the synthetic potential of the novel methodology. Gratifyingly, when subjecting to the reaction with (2-aminophenyl)(phenyl)methanone in a similar fashion, these differently substituted 3-acetyl-*N*-alkyl-2-chloroindoles were equally amenable to the reaction process, invariably leading to the formation of a series of structurally diverse indolo[2,3-*b*]quinolines (3b–3r) as expected in acceptable yields ranging 48–71% as listed in Table 2. According to the reaction results it appeared that the nature of the substituent did not exert an obvious influence on the product yields. What's more, there was no issue in using some highly substituted 3-acetyl-*N*-alkyl-2-chloroindoles for transformation to the corresponding indolo[2,3-*b*]quinoline products such as 3o–3r in comparable yields. Currently, our further investigations to expand the scope of the substrates 2-aminobenzophenones bearing different types of substitutions at the phenyl ring are being actively pursued in our laboratory and more studies on the evaluation of biological activity through structure–activity relationship are ongoing, which would be reported in future.

**Table 2:** Scope of 3-acetyl-*N*-alkyl-2-chloroindoles leading to indolo[2,3-*b*]quinolines^a^

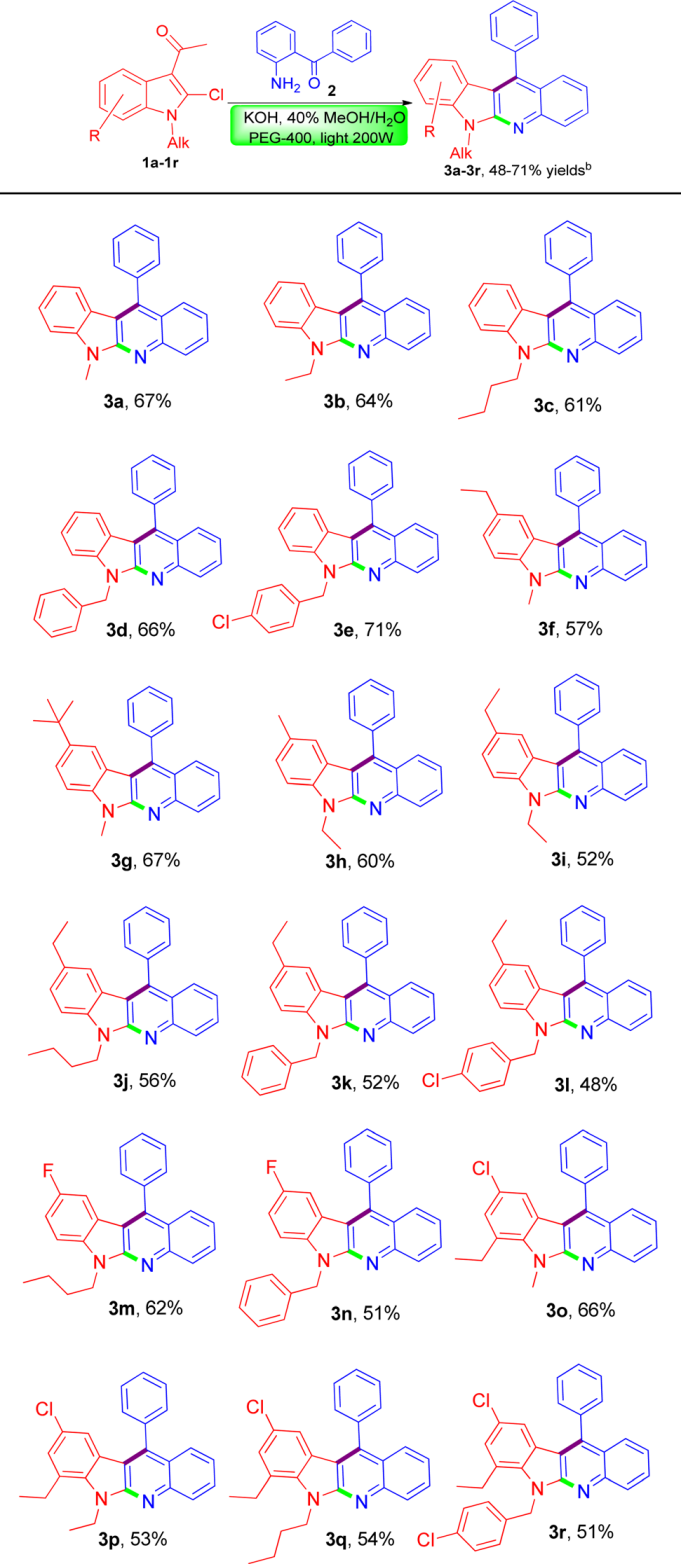

a Reaction conditions: a solution of 1 (0.5 mmol), 2 (0.55 mmol), KOH (0.56 g) and PEG-400 (100 mg, 50 mol%) in 40% methanol aqueous solution (5.0 mL) was irradiated by 200 Watt tungsten lamp.

b Isolated yields.

To the best of our knowledge, all these newly-synthesized compounds 3a–3r have never been reported, and their structures have been elucidated clearly on the basis of their spectroscopic data which are in good agreement with the proposed structures. Moreover, the obtained elemental analysis values are also in agreement with calculated data (see ESI†).

**Scheme 2:**
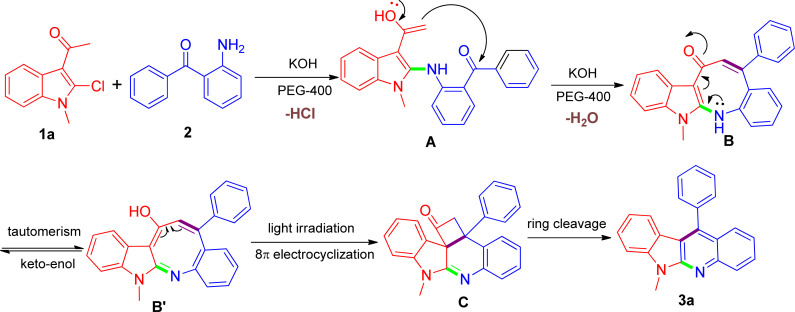
The proposed reaction mechanism for the synthesis of 3a.

Despite the unique reactivity behavior for the direct construction of indolo[2,3-*b*]quinolines in this work, we have not yet established the conclusive explanation at the current stage and the exact details regarding how the reaction proceed still remain unclear and under-studied. Herein, taking into consideration the entire outcome, a tentative proposition is given as shown in Scheme 2 using the model reaction as an example. We speculated that a putative azacyclooctotrienone B or its tautomer B′ might be constructed through a cascade reaction sequence, involving the nucleophilic substitution reaction of the amino group of 2 with the 2-chloro substituent on 1a affording the corresponding A, which was followed by *in situ* intramolecular aldol condensation reaction between the acetyl and the carbonyl with the presence of KOH. At this stage, we conjectured that the addition of PEG-400 might be able to facilitate the intermediate B arising due to its proper coordination with K^+^.^38^ We had made many attempts to isolate the intermediate but failed due to extreme instability of the structure during purification process. It has been observed that α,β-unsaturated carbonyl moiety could be easily photoexcited,^39^ and thus the intermediate B thereinto might be very prone to undergo thermal pericyclic reaction invoked by the help of light irradiation, leading to the generation of the transition state C. In this regard, there was a closely related photochemical pathway reported from Fahrenhorst-Jones and coworkers who discovered that the azacyclooctatriene obtained from an 8π-electrocyclisation reaction was perfectly placed to undergo a photochemical cyclisation under photochemical conditions leading to the formation of 2-azabicyclo[4.2.0]octa-4,7-diene,^40^ which was very similar to the transformation of B into C. Next, the inherent ring tension rendered the formed transition state C transient and amenable to strain-releasing reaction,^41^ resulting in the cleavage of the cyclobutanone ring and eventually leading to the formation of product 3a. Considering the fact that visible light irradiation involve in the reaction, we further designed a parallel experiment under the dark condition in order to gain some insight with regard to the possible reaction mechanism. Based on the experimental results we found that upon the same reaction was conducted under dark conditions, the reaction did not proceed satisfactorily, and the reaction mixture showed a combination of starting materials and numerous products, in which the desired product 3a was detected only in negligible amount that did not warrant isolation. Such results may adequately support our hypothesis that visible light might play an important role in the reaction. Although being consistent with the observed product and related literature precedents, our proposed mechanism still remains speculative until further experimentation can rigorously exclude alternative pathways. Our further investigations focusing on the detailed mechanistic studies by capturing key intermediates and providing adequate prove-of-concept are currently ongoing in our laboratory, and these results will be part of future reports.

## Conclusions

In summary, we have provided a straightforward, cost-effective and eco- and user-friendly route to easy access diverse range of *N*-alkyl- and 11-phenyl-modified indolo[2,3-*b*]quinoline system of the alkaloid neocryptolepine analogues. In comparison with those syntheses previously realized, this work not only represents a new simple and expeditious route, but also offers a practical and environmentally benign access to diversified indolo[2,3-*b*]quinoline derivatives. It possesses the attractive merits although the yields are moderate and there is still a demand for further investigations including the exploration of the reaction mechanism and the extension of the scope. In perspective, it is conceivable that this interesting reaction would be enough scope for further investigation and would likely render it a useful tool in the synthesis of this class of indolo[2,3-*b*]quinoline alkaloid derivatives. More studies toward the mechanism and the scope of this novel transformation are currently actively pursued in our laboratory.

## Data availability

The data supporting this article has been included in the ESI.†

## Author contributions

H. Z. completing the synthesis of the products; Y. J. completing the characteristic data recording and analysis; X. S. and X. W. conducting the synthesis of the products; T. L. writing the original draft, supervision and formal analysis; Y. L. project administration, conceptualization, reviewing & editing and funding acquisition. All authors read and approved the final version to be published.

## Conflicts of interest

There are no conflicts to declare.

## Supplementary Material

RA-014-D4RA05176A-s001

RA-014-D4RA05176A-s002
