# Supplementary material for: A new synthesis of indolo[2,3-b]quinolines from 3-acetyl-N-alkyl-2-chloroindoles with 2-aminobenzophenone
Source: RSC Adv. 2024 Sep 26;14(42):30707–12. doi: 10.1039/d4ra05176a (PMC11425041; doi:10.1039/d4ra05176a)
Supplement: RA-014-D4RA05176A-s001 [file RA-014-D4RA05176A-s001.pdf]

Supplementary Information (SI) for RSC Advances.
This journal is © The Royal Society of Chemistry 2024

A new synthesis of indolo[2,3-*b*]quinolines from 3-acetyl-N-alkyl-2- chloroindoles with 2-aminobenzophenone

Hong Zhang,[a] Yunhe Jiang,[b] Xiaoxue Sun,[b] Tianyu Liang,*[,b,c] Xiang Wang,[b] and Yang Li*[,b]

[a] *Experiment & Equipment Administration Center, Bohai University, Jinzhou 121000, P. R. China.*

[b] *College of Chemistry and Materials Engineering, Bohai University, Jinzhou 121000, P. R. China.*

[b] *Key Laboratory of Biotechnology and Bioresources Utilization of Ministry of Education, Dalian Minzu University, Dalian 116600, P. R. China.*

**E-mail: tyliang@bhu.edu.cn; bhuzh@163.com*

Supplementary Information

Table of Contents

1. General information 1

2. General procedure for synthesis of 3a-r...1

3. Characterization data of the synthesized products 3a-3r...1

4. ^{1}H and ^{13}C NMR spectra of compounds 3a-3r 8

1. General information

The chemicals used in this report were obtained from Energy Chemical and were used without further purification. Melting points were determined using a WRS-1B melting point apparatus. The IR spectra were obtained as KBr pellets in the range of 400–4000 cm^{-1} on a Shimadzu FTIR-8400S spectrophotometer (Shimadzu, Japan). The ^1H (400 MHz) and ^{13}C (100 MHz) NMR spectra were recorded on an Agilent 400-MR spectrometer using $CDCl_3$ as solvent. The reported chemical shifts (δ values) are given in parts per million downfield from tetramethylsilane (TMS) as the internal standard (NMR abbreviations: s = singlet, d = doublet, t = triplet, q = quartet, dd = doublet of doublets, dt = double triplet, td = triplet double, m = multiplet, J = coupling constant). Elemental analyses were carried out on an EA 2400II elemental analyzer (PerkinElmer, Waltham, MA). X-ray diffraction analysis data were collected with a Bruker Smart Apex CCD diffractometer using Mo Kα radiation (λ =0.071 073 nm) at 293 K. The reaction progress was monitored by thin-layer chromatography (TLC) on silica gel GF254 plates using UV light as the visualizing agent, and the products were purified by flash column chromatography on silica gel (200-300 meshes) from the Qingdao Marine Chemical Factory in China using petroleum ether/ethyl acetate (1 : 20) as eluent.

2. General procedure for synthesis of 6-alkyl-11-phenyl-6*H*-indolo[2,3-*b*]quinolines (3a-r)

The respective 3-acetyl-N-alkyl-2-chloroindoles (**1a-1r**, 0.5 mmol) and KOH (10 mmol, 560 mg) were first dissolved completely in 40% methanol aqueous solution (5 mL) under stirring. Subsequently, 2-aminobenzophenone (5.5 mmol, 108 mg) and PEG-400 (100 mg, 50 mol%) were added respectively to the above mixture and heated with stirring to boil gently. And then the resulting reaction mixture was exposed to illumination with a 200W tungsten lamp. The conversion was monitored by TLC. After the reaction was complete within 8-12 h, the mixture was poured slowly with stirring into cold water (15 mL) followed by neutralization with 1 M HCl solution in which the generated yellow precipitates were observed. The formed crude products were collected and purified by flash chromatography to give the corresponding compounds **3a-3r** as yellow solids. Their yields, physical properties and the spectral and analytical data are given below.

3. Characterization data of the synthesized products 3a-3r

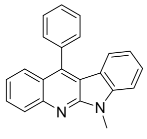

6-Methyl-11-phenyl-6*H*-indolo[2,3-*b*]quinoline (3a): Yield 64%, mp 158-159 °C, IR (KBr): 3058, 2955, 1583, 1499, 1467, 1392, 1274; ^1H NMR ($CDCl_3$, 400 MHz): 4.05 (s, 3H, Me-H), 7.02 (t, *J* = 7.6 Hz, 1H, Ar-H), 7.08 (d, *J* = 8.0 Hz, 1H, Ar-H), 7.37 (d, *J* = 8.0 Hz, 1H, Ar-H), 7.41 (d, *J* = 8.0 Hz, 1H, Ar-H), 7.50 (d, *J* = 8.0 Hz, 1H, Ar-H), 7.55 (dd, *J* = 7.6, 2.0 Hz, 2H, Ar-H), 7.61-

7.68 (m, 3H, Ar-H), 7.71-7.76 (m, 2H, Ar-H), 8.22 (d, *J* = 8.4 Hz, 1H, Ar-H); ^{13}C NMR ($CDCl_3$, 100 MHz): 27.84, 108.44, 116.01, 119.78, 120.50, 122.83, 122.99, 123.65, 126.39, 127.31, 127.37, 127.78, 128.52, 128.83, 128.95, 129.30, 136.43, 142.43, 142.83. Anal. Calcd for $C_{22}H_{16}N_2$: C, 85.69; H, 5.23; N, 9.08. Found: C, 85.92; H, 5.05; N, 9.01.

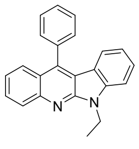

6-Ethyl-11-phenyl-6H-indolo[2,3-*b*]quinoline (3b): Yield 68%, mp 175-176 °C, IR (KBr): 3043, 2960, 1578, 1472, 1448, 1366, 1264; ^{1}H NMR ($CDCl_3$, 400 MHz): 1.56 (t, *J* = 7.2 Hz, 3H, Et-H), 4.68 (q, *J* = 6.8 Hz, 2H, Et-H), 7.01 (t, *J* = 7.6 Hz, 1H, Ar-H), 7.08 (d, *J* = 7.6 Hz, 1H, Ar-H), 7.38 (t, *J* = 8.0 Hz, 1H, Ar-H), 7.43 (d, *J* = 7.6 Hz, 1H, Ar-H), 7.50 (t, *J* = 7.6 Hz, 1H, Ar-H), 7.54 (dd, *J* = 7.6, 1.6 Hz, 2H, Ar-H), 7.61-7.67 (m, 3H, Ar-H), 7.75 (t, *J* = 8.0 Hz, 2H, Ar-H), 8.21 (d, *J* = 7.6 Hz, 1H, Ar-H); 13C NMR ($CDCl_3$, 100 MHz): 13.71, 36.11, 108.58, 116.01, 119.53, 120.70, 122.72, 123.16, 123.68, 126.37, 127.47, 127.51, 127.53, 127.56, 127.67, 128.48, 128.67, 128.96, 129.32, 136.56, 141.86. Anal. Calcd for $C_{23}H_{18}N_2$: C, 85.68; H, 5.63; N, 8.69. Found: C, 85.39; H, 5.52; N, 8.75.

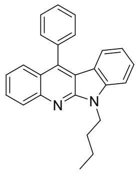

6-Butyl-11-phenyl-6*H*-indolo[2,3-*b*]quinoline (3c): Yield 61%, mp 156-158 °C, IR (KBr): 3046, 2944, 1598, 1483, 1455, 1369, 1275; ^{1}H NMR ($CDCl_3$, 400 MHz): 1.01 (t, *J* = 7.6 Hz, 3H, *n*-Bu), 1.50 (sext, *J* = 7.6 Hz, 2H, *n*-Bu), 1.98 (quint, *J* = 7.6 Hz, 2H, *n*-Bu), 4.59 (t, *J* = 7.6 Hz, 2H, *n*-Bu), 6.99 (t, *J* = 7.6 Hz, 1H, Ar-H), 7.08 (d, *J* = 7.6 Hz, 1H, Ar-H), 7.36 (t, *J* = 7.6 Hz, 1H, Ar-H), 7.43 (d, *J* = 8.0 Hz, 1H, Ar-H), 7.48 (t, *J* = 7.6 Hz, 1H, Ar-H), 7.55 (dd, *J* = 7.6, 2.0 Hz, 2H, Ar-H), 7.61-7.68 (m, 3H, Ar-H), 7.71 (d, *J* = 8.0 Hz, 1H, Ar-H), 7.75 (d, *J* = 8.0 Hz, 1H, Ar-H), 8.19 (d, *J* = 8.0 Hz, 1H, Ar-H); ^{13}C NMR ($CDCl_3$, 100 MHz): 13.92, 20.38, 30.63, 41.16, 108.75, 119.34, 119.41, 120.60, 122.64, 123.09, 123.62, 123.65, 126.32, 127.58, 127.68, 128.45, 128.53, 128.59, 128.94, 129.33, 136.62, 142.28. Anal. Calcd for $C_{25}H_{22}N_2$: C, 85.68; H, 6.33; N, 7.99. Found: C, 85.89; H, 6.47; N, 7.84.

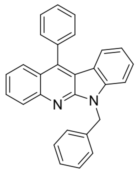

6-Benzyl-11-phenyl-6*H*-indolo[2,3-*b*]quinoline (3d): Yield 66%, mp 168-169 °C, IR (KBr): 3051, 2951, 1598, 1479, 1476, 1383, 1266; ^{1}H NMR ($CDCl_3$, 400 MHz): 5.83 (s, 2H, CH_2Ph-H), 6.99 (t, *J* = 7.6 Hz, 1H, Ar-H), 7.08 (d, *J* = 7.6 Hz, 1H, Ar-H), 7.22-7.30 (m, 4H, Ar-H), 7.37-7.41

(m, 4H, Ar-H), 7.57 (d, *J* = 7.6 Hz, 2H, Ar-H), 7.62-7.69 (m, 3H, Ar-H), 7.73 (d, *J* = 8.0 Hz, 1H, Ar-H), 7.77 (d, *J* = 8.4 Hz, 1H, Ar-H), 8.19 (d, *J* = 8.0 Hz, 1H, Ar-H); ^{13}C NMR ($CDCl_3$, 100 MHz): 44.97, 109.42, 115.90, 119.93, 120.77, 122.96, 123.04, 123.93, 126.37, 127.23, 127.34, 127.74, 128.55, 128.62, 128.76, 128.98, 129.32, 136.46, 137.20, 142.05. Anal. Calcd for $C_{28}H_{20}N_2$: C, 87.47; H, 5.24; N, 7.29. Found: C, 87.76; H, 5.04; N, 7.18.

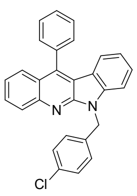

6-(4-Chlorobenzyl)-11-phenyl-6*H*-indolo[2,3-*b*]quinoline (3e): Yield 71%, mp 197-198 ^{o}C, IR (KBr): 3060, 2956, 1592, 1479, 1455, 1379, 1264; ^{1}H NMR ($CDCl_3$, 400 MHz): 5.78 (s, 2H, CH_2Ph-H), 7.01 (t, *J* = 7.6 Hz, 1H, Ar-H), 7.09 (d, *J* = 7.6 Hz, 1H, Ar-H), 7.23-7.26 (m, 3H, Ar-H), 7.32 (d, *J* = 8.4 Hz, 2H, Ar-H), 7.41 (t, *J* = 7.6 Hz, 2H, Ar-H), 7.56 (dd, *J* = 7.2, 2.0 Hz, 2H, Ar-H), 7.62-7.69 (m, 3H, Ar-H), 7.73 (d, *J* = 7.6 Hz, 1H, Ar-H), 7.77 (d, *J* = 8.4 Hz, 1H, Ar-H), 8.19 (d, *J* = 8.4 Hz, 1H, Ar-H); ^{13}C NMR ($CDCl_3$, 100 MHz): 44.37, 109.20, 115.84, 120.10, 120.15, 120.84, 123.06, 123.15, 124.00, 126.41, 127.74, 127.79, 128.60, 128.66, 128.80, 128.84, 129.01, 129.20, 129.29, 133.16, 135.74, 136.36, 141.81. Anal. Calcd for $C_{28}H_{19}ClN_2$: C, 80.28; H, 4.57; N, 6.69. Found: C, 80.51; H, 4.82; N, 6.54.

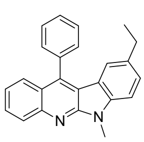

9-Ethyl-6-methyl-11-phenyl-6*H*-indolo[2,3-*b*]quinoline (3f): Yield 57%, mp 164-166 ^{o}C, IR (KBr): 3066, 2960, 1589, 1473, 1455, 1376, 1250; ^{1}H NMR ($CDCl_3$, 400 MHz): 1.12 (t, *J* = 7.6 Hz, 3H, CH_2CH_3), 2.58 (q, *J* = 7.6 Hz, 2H, CH_2CH_3), 4.03 (s, 3H, CH_3), 6.87 (s, 1H, ArH), 7 .31 (d, *J* = 8.4 Hz, 1H, ArH), 7.36 (t, *J* = 8.0 Hz, 2H, ArH), 7.54 (d, *J* = 7.6 Hz, 2H, ArH), 7.62-7.66 (m, 3H, ArH), 7.72 (t, *J* = 8.0 Hz, 1H, ArH), 7.76 (d, *J* = 8.4 Hz, 1H, ArH), 8.19 (d, *J* = 8.0 Hz, 1H, ArH). ^{13}C NMR ($CDCl_3$, 100 MHz): 15.88, 27.85, 28.60, 108.20, 120.55, 121.95, 122.68, 123.55, 126.38, 127.33, 127.38, 127.81, 128.49, 128.70, 128.71, 128.73, 128.75, 128.86, 129.37, 135.68, 136.50, 141.19. Anal. Calcd for $C_{24}H_{20}N_2$: C, 85.68; H, 5.99; N, 8.33. Found: C, 85.39; H, 6.21; N, 8.12.

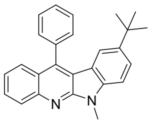

9-(*tert*-Butyl)-6-methyl-11-phenyl-6*H*-indolo[2,3-*b*]quinoline (3g): Yield 67%, mp 211-212 ^{o}C, IR (KBr): 3062, 2965, 1569, 1482, 1456, 1387, 1271; ^{1}H NMR ($CDCl_3$, 400 MHz): 1.19 (s, 9H, n-Bu), 4.02 (s, 3H, Me-H), 7.06 (d, *J* = 1.2 Hz, 1H, Ar-H), 7.34 (d, *J* = 8.8 Hz, 1H, Ar-H), 7.39 (t, *J* = 7.6 Hz, 1H, Ar-H), 7.54-7.57 (m, 3H, Ar-H), 7.65 (d, *J* = 8.0 Hz, 2H, Ar-H), 7.69 (d, *J* = 8.8 Hz,

1H, Ar-H), 7.74 (t, *J* = 7.6 Hz, 1H, Ar-H), 7.82 (d, *J* = 8.4 Hz, 1H, Ar-H), 8.21 (d, *J* = 8.4 Hz, 1H, Ar-H); ^{13}C NMR ($CDCl_3$, 100 MHz): 27.76, 31.56, 34.42, 107.87, 116.33, 119.66, 120.16, 122.64, 123.51, 125.29, 126.35, 127.43, 128.43, 128.61, 128.85, 129.43, 136.57, 140.90, 142.11, 142.64, 146.62, 152.66. Anal. Calcd for $C_{26}H_{24}N_2$: C, 85.68; H, 6.64; N, 7.69. Found: C, 85.37; H, 6.80; N, 7.99.

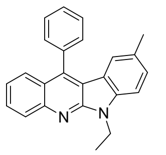

6-Ethyl-9-methyl-11-phenyl-6*H*-indolo[2,3-*b*]quinoline (3h): Yield 60%, mp 196-197 ^oC, IR (KBr): 3047, 2943, 1578, 1489, 1448, 1370, 1262; ^{1}H NMR ($CDCl_3$, 400 MHz): 1.54 (t, *J* = 7.2 Hz, 3H, Et-H), 2.28 (s, 3H, CH3), 4.66 (q, *J* = 7.2 Hz, 2H, Et-H), 6.83 (s, 1H, Ar-H), 7.29-7.37 (m, 3H, Ar-H), 7.53 (dd, *J* = 7.6, 2.0 Hz, 2H, Ar-H), 7.61-7.66 (m, 3H, Ar-H), 7.73 (t, *J* = 8.4 Hz, 2H, Ar-H), 8.18 (d, *J* = 8.4 Hz, 1H, Ar-H); ^{13}C NMR ($CDCl_3$, 100 MHz): 13.70, 21.37, 36.07, 108.30, 115.97, 120.76, 122.54, 123.38, 123.62, 126.37, 127.53, 128.45, 128.56, 128.69, 128.77, 128.90, 129.34, 136.64, 139.98, 142.09, 146.67, 151.86. Anal. Calcd for $C_{24}H_{20}N_2$: C, 85.68; H, 5.99; N, 8.33. Found: C, 85.85; H, 5.85; N, 8.56.

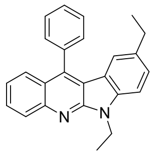

6,9-Diethyl-11-phenyl-6*H*-indolo[2,3-*b*]quinoline (3i): Yield 52%, mp 191-193 ^oC, IR (KBr): 3059, 2959, 1587, 1476, 1459, 1347, 1275; ^{1}H NMR ($CDCl_3$, 400 MHz): 1.13 (t, *J* = 7.6 Hz, 3H, CH2CH3), 1.53 (t, *J* = 7.2 Hz, 3H, CH2CH3), 2.59 (q, *J* = 7.6 Hz, 2H, CH2CH3), 4.63 (q, *J* = 7.2 Hz, 2H, CH2CH3), 6.87 (s, 1H, ArH), 7.34-7.38 (m, 3H, ArH), 7.55 (d, *J* = 7.6 Hz, 2H, ArH), 7.62-7.68 (m, 3H, ArH), 7.72 (t, *J* = 8.0 Hz, 1H, ArH), 7.76 (d, *J* = 8.4 Hz, 1H, ArH), 8.18 (d, *J* = 8.4 Hz, 1H, ArH); ^{13}C NMR ($CDCl_3$, 100 MHz): 13.75, 15.87, 28.59, 36.13, 108.36, 116.13, 120.75, 122.10, 122.59, 123.56, 126.36, 127.45, 127.72, 128.47, 128.60, 128.88, 129.38, 135.41, 136.61, 140.17. Anal. Calcd for $C_{25}H_{22}N_2$: C, 85.68; H, 6.33; N, 7.99. Found: C, 85.40; H, 6.44; N, 8.28.

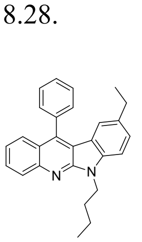

6-Butyl-9-ethyl-11-phenyl-6*H*-indolo[2,3-*b*]quinoline (3j): Yield 56%, mp 96-99 ^oC, IR (KBr): 3047, 2943, 1578, 1489, 1456, 1383, 1274; ^{1}H NMR ($CDCl_3$, 400 MHz): 1.02 (td, *J* = 7.2, 1.2 Hz, 3H, *n*-Bu), 1.15 (td, *J* = 7.6, 1.6 Hz, 3H, Et-H), 1.53 (sext, *J* = 7.6 Hz, 2H, *n*-Bu), 2.00 (quint, *J* = 7.6 Hz, 2H, *n*-Bu), 2.61 (q, *J* = 7.6 Hz, 2H, Et-H), 4.58 (t, *J* = 7.6 Hz, 2H, *n*-Bu), 6.87 (s, 1H, Ar-

H), 7.33 (d, J = 8.4 Hz, 2H, Ar-H), 7.37 (d, J = 7.2 Hz, 1H, Ar-H), 7.53-7.56 (m, 2H, Ar-H), 7.61-7.67 (m, 3H, Ar-H), 7.72 (t, J = 7.6 Hz, 1H, Ar-H), 7.77 (d, J = 8.4 Hz, 1H, Ar-H), 8.18 (d, J = 8.4 Hz, 1H, Ar-H); ^{13}C NMR ($CDCl_3$, 100 MHz): 13.93, 15.81, 20.38, 28.56, 30.67, 41.14, 108.52, 120.63, 122.00, 122.43, 122.49, 123.52, 126.32, 127.63, 127.74, 128.38, 128.43, 128.55, 128.85, 129.38, 135.26, 136.65, 140.58. Anal. Calcd for $C_{27}H_{26}N_2$: C, 85.68; H, 6.92; N, 7.40. Found: C, 86.01; H, 6.67; N, 7.28.

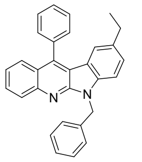

6-Benzyl-9-ethyl-11-phenyl-6*H*-indolo[2,3-*b*]quinoline (3k): Yield 52%, mp 130-131 ^{o}C, IR (KBr): 3061, 2954, 1579, 1489, 1436, 1371, 1264; ^{1}H NMR ($CDCl_3$, 400 MHz): 1.11 (t, J = 7.6 Hz, 3H, Et-H), 2.58 (q, J = 7.6 Hz, 2H, Et-H), 5.80 (s, 2H, CH_2Ph-H), 6.87 (s, 1H, Ar-H), 7.18-7.25 (m, 3H, Ar-H), 7.30 (d, J = 7.6 Hz, 2H, Ar-H), 7.36-7.40 (m, 3H, Ar-H), 7.58 (d, J = 7.6 Hz, 2H, Ar-H), 7.63-7.67 (m, 3H, Ar-H), 7.74 (t, J = 7.6 Hz, 1H, Ar-H), 7.79 (d, J = 8.4 Hz, 1H, Ar-H), 8.19 (d, J = 8.0 Hz, 1H, Ar-H); ^{13}C NMR ($CDCl_3$, 100 MHz): 15.71, 28.54, 44.98, 109.18, 111.81, 114.80, 120.81, 121.97, 122.80, 123.81, 126.36, 127.23, 127.29, 127.68, 127.79, 128.53, 128.60, 128.67, 128.89, 133.64, 135.77, 136.51, 137.41, 140.35, 142.61. Anal. Calcd for $C_{30}H_{24}N_2$: C, 87.35; H, 5.86; N, 6.79. Found: C, 87.69; H, 5.56; N, 6.81.

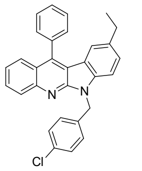

6-(4-Chlorobenzyl)-9-ethyl-11-phenyl-6*H*-indolo[2,3-*b*]quinoline (3l): Yield 48%, mp 154-157 ^{o}C, IR (KBr): 3066, 2967, 1585, 1492, 1479, 1376, 1265; ^{1}H NMR ($CDCl_3$, 400 MHz): 1.12 (t, J = 7.6 Hz, 3H, Et-H), 2.58 (q, J = 7.6 Hz, 2H, Et-H), 5.76 (s, 2H, CH_2Ph-H), 6.88 (s, 1H, Ar-H), 7.16 (d, J = 8.0 Hz, 1H, Ar-H), 7.23-7.26 (m, 3H, Ar-H), 7.32 (d, J = 8.4 Hz, 2H, Ar-H), 7.40 (t, J = 7.6 Hz, 1H, Ar-H), 7.57 (dd, J = 7.6, 2.4 Hz, 2H, Ar-H), 7.65-7.69 (m, 3H, Ar-H), 7.74 (t, J = 7.6 Hz, 1H, Ar-H), 7.79 (d, J = 8.4 Hz, 1H, Ar-H), 8.18 (d, J = 8.0 Hz, 1H, Ar-H); ^{13}C NMR ($CDCl_3$, 100 MHz): 15.71, 28.54, 44.43, 108.98, 120.85, 122.07, 122.92, 122.97, 123.00, 123.86, 126.40, 127.86, 127.90, 127.94, 128.60, 128.65, 128.76, 128.92, 129.34, 130.85, 133.09, 135.83, 136.02, 136.39, 140.07. Anal. Calcd for $C_{30}H_{23}ClN_2$: C, 80.61; H, 5.19; N, 6.27. Found: C, 80.46; H, 5.12; N, 6.45.

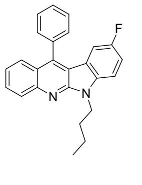

6-Butyl-9-fluoro-11-phenyl-6*H*-indolo[2,3-*b*]quinoline (3m): Yield 62%, mp 148-150 ^{o}C, IR (KBr): 3058, 2970, 1578, 1485, 1437, 1383, 1270; ^{1}H NMR ($CDCl_3$, 400 MHz): 1.03 (t, *J* = 7.6 Hz, 3H, *n*-Bu), 1.52 (sext, *J* = 7.6 Hz, 2H, *n*-Bu), 1.99 (quint, *J* = 7.6 Hz, 2H, *n*-Bu), 4.58 (t, *J* = 7.6 Hz, 2H, *n*-Bu), 6.74 (dd, *J* = 7.2, 2.4 Hz, 1H, Ar-H), 7.22 (td, *J* = 7.2, 2.4 Hz, 1H, Ar-H), 7.32 (t, *J* = 8.4 Hz, 1H, Ar-H), 7.37 (t, *J* = 7.6 Hz, 1H, Ar-H), 7.53 (dd, *J* = 7.6, 2.0 Hz, 2H, Ar-H), 7.64-7.74 (m, 5H, Ar-H), 8.18 (d, *J* = 8.0 Hz, 1H, Ar-H); ^{13}C NMR ($CDCl_3$, 100 MHz): 13.92, 20.38, 30.63, 41.27, 109.15 (*J* = 32 Hz), 109.24, 109.49, 114.77, 115.01, 121.04, 121.14, 122.85 (*J* = 28 Hz), 123.44, 126.46, 127.74 (*J* = 28 Hz), 128.77, 128.96, 129.10, 129.17, 136.06, 138.51, 140.08. Anal. Calcd for $C_{25}H_{21}FN_2$: C, 81.50; H, 5.74; N, 7.60. Found: C, 81.71; H, 5.81; N, 7.33.

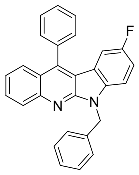

6-Benzyl-9-fluoro-11-phenyl-6*H*-indolo[2,3-*b*]quinoline (3n): Yield 57%, mp 195-197 ^{o}C, IR (KBr): 3061, 2913, 1595, 1479, 1449, 1395, 1290; ^{1}H NMR ($CDCl_3$, 400 MHz): 5.81 (s, 2H, CH_2Ph-H), 6.75 (dd, *J* = 8.4, 2.0 Hz, 1H, Ar-H), 7.12 (td, *J* = 8.8, 2.4 Hz, 1H, Ar-H), 7.19 (dd, *J* = 8.4, 4.4 Hz, 1H, Ar-H), 7.26 (s, 1H, Ar-H), 7.31 (t, *J* = 8.0 Hz, 2H, Ar-H), 7.35 (d, *J* = 7.6 Hz, 2H, Ar-H), 7.40 (t, *J* = 8.0 Hz, 1H, Ar-H), 7.55 (dd, *J* = 7.6, 2.0 Hz, 2H, Ar-H), 7.63-7.70 (m, 3H, Ar-H), 7.74 (d, *J* = 7.6 Hz, 1H, Ar-H), 7.77 (d, *J* = 8.0 Hz, 1H, Ar-H), 8.19 (d, *J* = 8.4 Hz, 1H, Ar-H); ^{13}C NMR ($CDCl_3$, 100 MHz): 45.08, 109.25, 109.50, 109.81, 109.88 (*J* = 28Hz), 114.91, 115.51, 121.28, 121.37 (*J* = 36 Hz), 123.07, 123.73, 126.48, 127.17, 127.45, 128.69, 128.84, 129.12, 129.16 (*J* = 16 Hz), 135.90, 137.04, 138.24, 156.13, 158.48. Anal. Calcd for $C_{28}H_{19}FN_2$: C, 83.56; H, 4.76; N, 6.96. Found: C, 83.68; H, 4.69; N, 7.07.

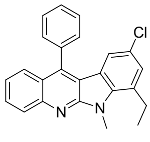

9-Chloro-7-ethyl-6-methyl-11-phenyl-6*H*-indolo[2,3-*b*]quinoline (3o): Yield 66%, mp 209-210 ^{o}C, IR (KBr): 3025, 2967, 1578, 1485, 1454, 1379, 1294, 1123; ^{1}H NMR ($CDCl_3$, 400 MHz): 1.41 (t, *J* = 7.6 Hz, 3H, Et-H), 3.22 (q, *J* = 7.6 Hz, 2H, Et-H), 4.31 (s, 3H, Me-H), 6.80 (d, *J* = 1.2 Hz, 1H, Ar-H), 7.22 (d, *J* = 1.6 Hz, 1H, Ar-H), 7.38 (t, *J* = 7.6 Hz, 1H, Ar-H), 7.48 (dd, *J* = 7.6, 2.4 Hz, 2H, Ar-H), 7.65-7.74 (m, 5H, Ar-H), 8.18 (d, *J* = 8.4 Hz, 1H, Ar-H); ^{13}C NMR ($CDCl_3$, 100 MHz): 16.59, 25.48, 30.81, 114.97, 120.47, 122.70, 122.94, 123.83, 124.92, 126.44, 127.60, 128.39, 128.75, 128.88, 128.99, 129.11, 136.03, 139.08, 142.65, 146.90, 153.04. Anal. Calcd for $C_{24}H_{19}ClN_2$: C, 77.72; H, 5.16; N, 7.55. Found: C, 77.86; H, 5.19; N, 7.37.

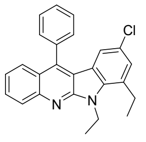

9-Chloro-6,7-diethyl-11-phenyl-6*H*-indolo[2,3-*b*]quinoline (3p): Yield 53%, mp 167-168 °C, IR (KBr): 3049, 2922, 1573, 1487, 1431, 1402, 1296; ^{1}H NMR ($CDCl_3$, 400 MHz): 1.41 (t, *J* = 7.6 Hz, 3H, Et-H), 1.48 (t, *J* = 7.2 Hz, 3H, Et-H), 3.15 (q, *J* = 7.6 Hz, 2H, Et-H), 4.88 (q, *J* = 7.2 Hz, 2H, Et-H), 6.82 (d, *J* = 2.0 Hz, 1H, Ar-H), 7.23 (d, *J* = 2.0 Hz, 1H, Ar-H), 7.37 (t, *J* = 7.6 Hz, 1H, Ar-H), 7.49 (dd, *J* = 7.6, 2.4 Hz, 2H, Ar-H), 7.63-7.73 (m, 5H, Ar-H), 8.18 (d, *J* = 8.4 Hz, 1H, Ar-H); ^{13}C NMR ($CDCl_3$, 100 MHz): 15.31, 16.01, 25.56, 37.96, 115.20, 120.59, 122.91, 123.11, 123.91, 124.87, 126.41, 127.78, 127.98, 128.73, 128.78, 128.87, 129.11, 136.10, 137.96, 142.49, 147.02, 152.59. Anal. Calcd for $C_{25}H_{21}ClN_2$: C, 78.01; H, 5.50; N, 7.28. Found: C, 78.27; H, 5.69; N, 6.99.

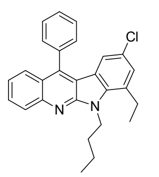

6-Butyl-9-chloro-7-ethyl-11-phenyl-6*H*-indolo[2,3-*b*]quinoline (3q): Yield 54%, mp 169-170 °C, IR (KBr): 3047, 2943, 1597, 1481, 1453, 1377, 1267; ^{1}H NMR ($CDCl_3$, 400 MHz): 1.01 (t, *J* = 7.6 Hz, 3H, *n*-Bu), 1.39 (t, *J* = 7.6 Hz, 3H, Et-H), 1.50 (sext, *J* = 7.6 Hz, 2H, *n*-Bu), 1.85 (quint, *J* = 7.6 Hz, 2H, *n*-Bu), 3.12 (q, J = 7.6 Hz, 2H, Et-H), 4.78 (t, *J* = 7.6 Hz, 2H, *n*-Bu), 6.81 (d, *J* = 2.0 Hz, 1H, Ar-H), 7.23 (d, *J* = 2.0 Hz, 1H, Ar-H), 7.37 (t, *J* = 7.6 Hz, 1H, Ar-H), 7.50 (dd, *J* = 7.6, 2.0 Hz, 2H, Ar-H), 7.62-7.67 (m, 3H, Ar-H), 7.73 (td, *J* = 8.4, 2.0 Hz, 2H, Ar-H), 8.18 (d, *J* = 8.4 Hz, 1H, Ar-H); ^{13}C NMR ($CDCl_3$, 100 MHz): 13.90, 16.05, 20.08, 25.60, 32.31, 42.88, 115.02, 120.57, 122.87, 123.08, 123.88, 124.84, 126.38, 127.83, 128.08, 128.71, 128.80, 128.81, 129.10, 129.13, 136.12, 138.16, 142.43, 146.98, 152.97. Anal. Calcd for $C_{27}H_{25}ClN_2$: C, 78.53; H, 6.10; N, 6.78. Found: C, 78.69; H, 6.17; N, 6.57.

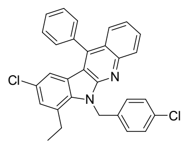

9-Chloro-6-(4-chlorobenzyl)-7-ethyl-11-phenyl-6*H*-indolo[2,3-*b*]quinoline (3r): Yield 51%, mp 184-185 °C, IR (KBr): 3055, 2970, 1585, 1479, 1429, 1360, 1256; ^{1}H NMR ($CDCl_3$, 400 MHz): 1.23 (t, *J* = 7.6 Hz, 3H, Et-H), 2.87 (q, *J* = 7.6 Hz, 2H, Et-H), 6.01 (s, 2H, CH_2Ph-H), 6.85 (d, *J* = 1.2 Hz, 1H, Ar-H), 6.97 (d, *J* = 8.0 Hz, 1H, Ar-H), 7.16 (d, *J* = 1.6 Hz, 1H, Ar-H), 7.22 (d, *J* = 8.0 Hz, 1H, Ar-H), 7.37 (t, *J* = 7.6 Hz, 1H, Ar-H), 7.52 (dd, *J* = 7.2, 1.2 Hz, 2H, Ar-H), 7.66-7.72 (m, 5H, Ar-H), 8.10 (d, *J* = 8.4 Hz, 1H, Ar-H); ^{13}C NMR ($CDCl_3$, 100 MHz): 16.10, 25.01, 45.93, 114.71, 120.63, 123.10, 123.23, 124.26, 125.52, 126.43, 127.09, 127.87, 128.29, 128.86, 128.93, 129.05, 129.13, 129.16, 132.86, 135.93, 137.34, 138.10, 142.91, 147.03, 152.90. Anal. Calcd for $C_{30}H_{22}Cl_2N_2$: C, 74.85; H, 4.61; N, 5.82. Found: C, 74.60; H, 4.68; N, 6.10.

4. ^{1}H and ^{13}C NMR spectra of compounds 3a-3r

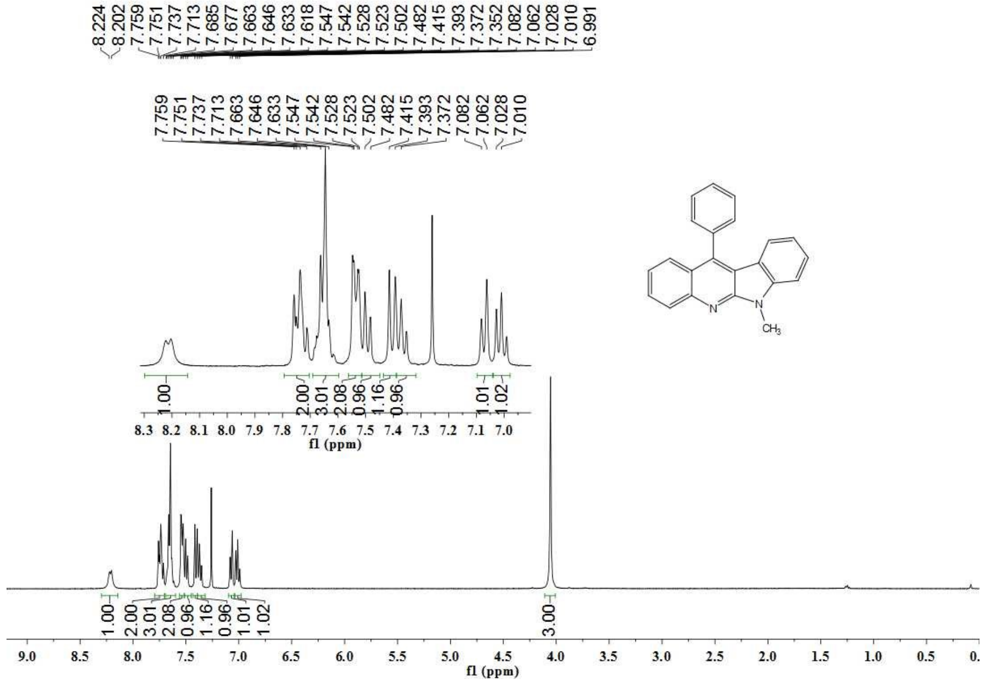

Fig. S1 ^{1}H NMR spectrum of **3a**

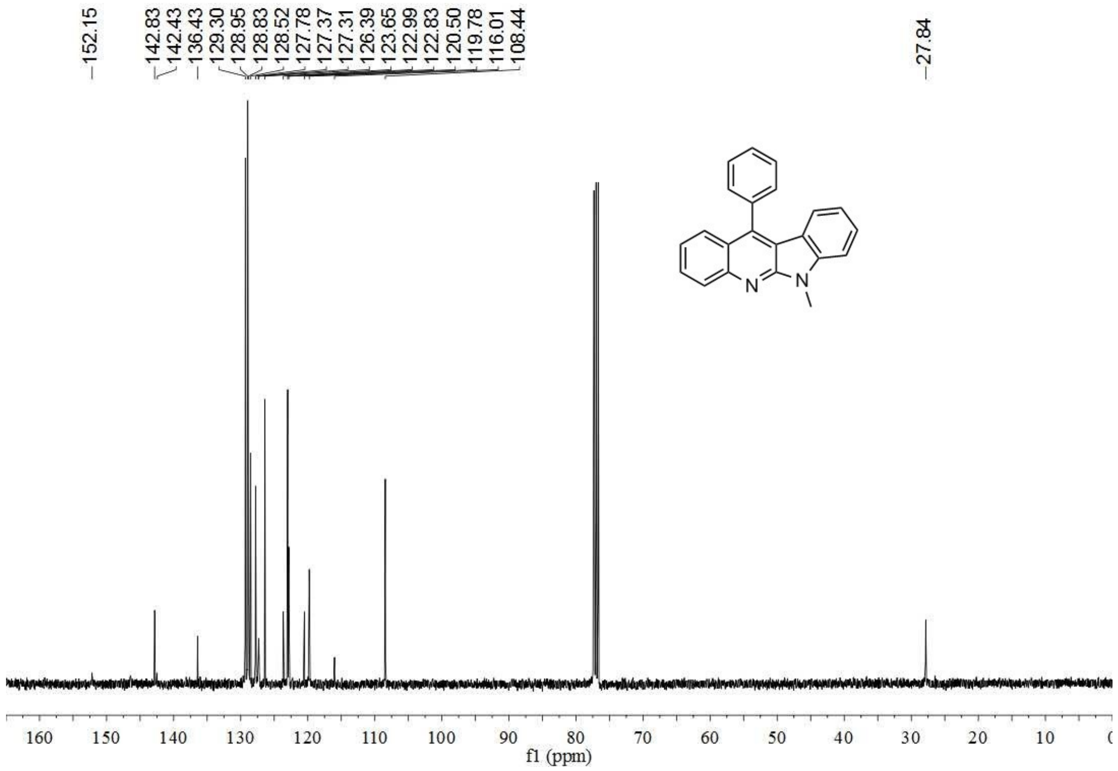

Fig. S2 ^{13}C NMR spectrum of **3a**

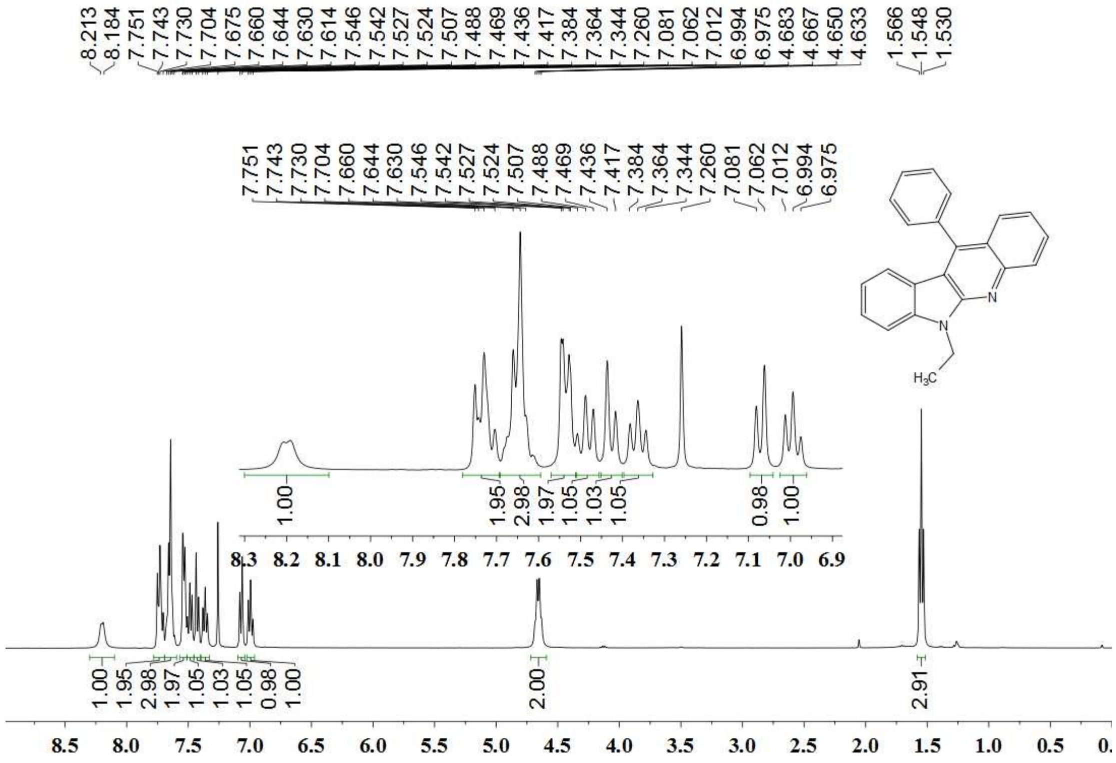

Fig. S3 ^{1}H NMR spectrum of **3b**

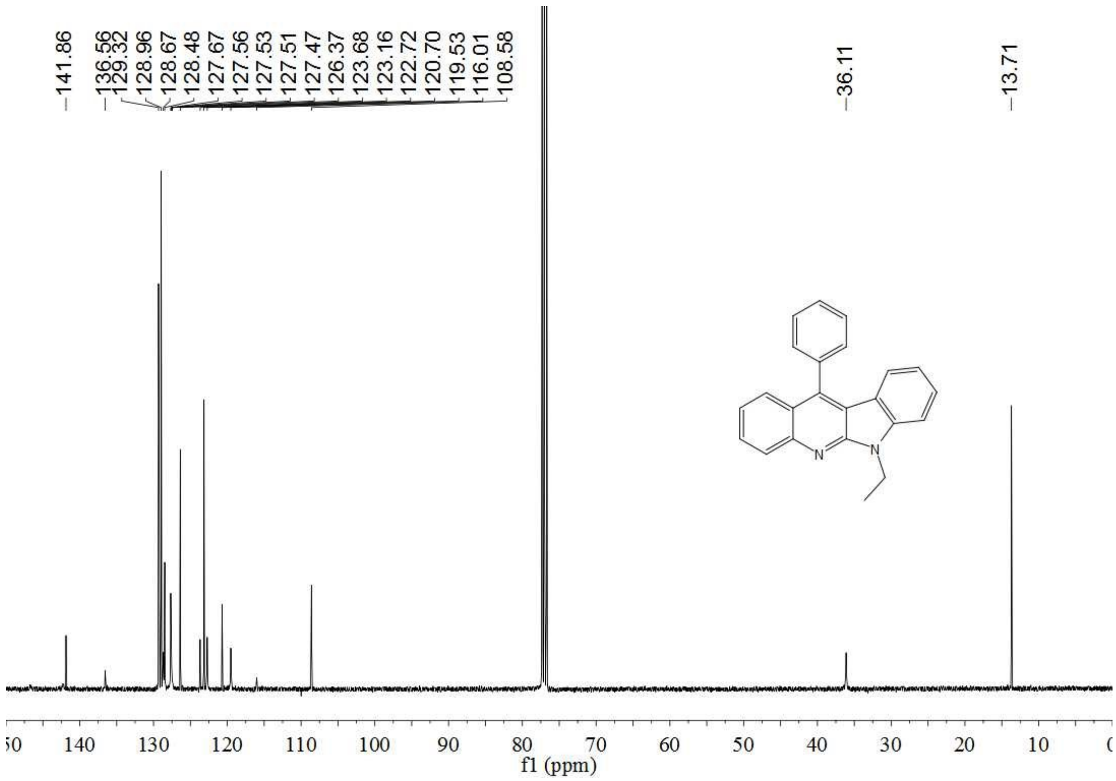

Fig. S4 ^{13}C NMR spectrum of **3b**

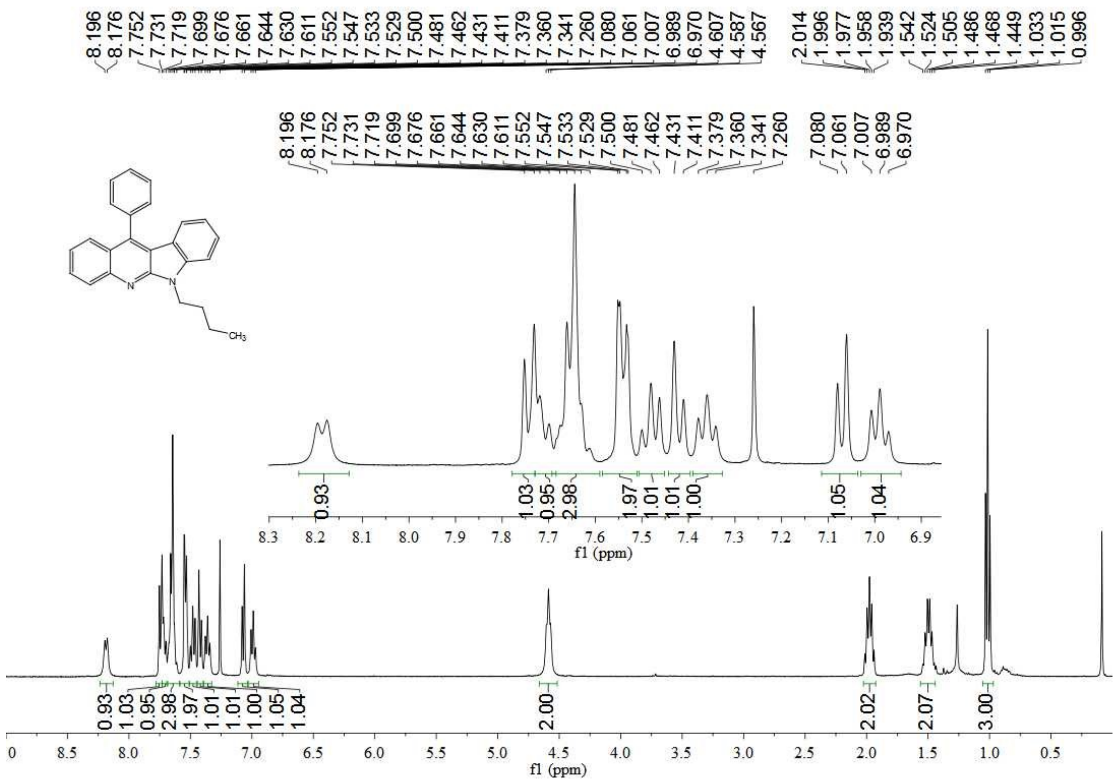

Fig. S5 ^{1}H NMR spectrum of **3c**

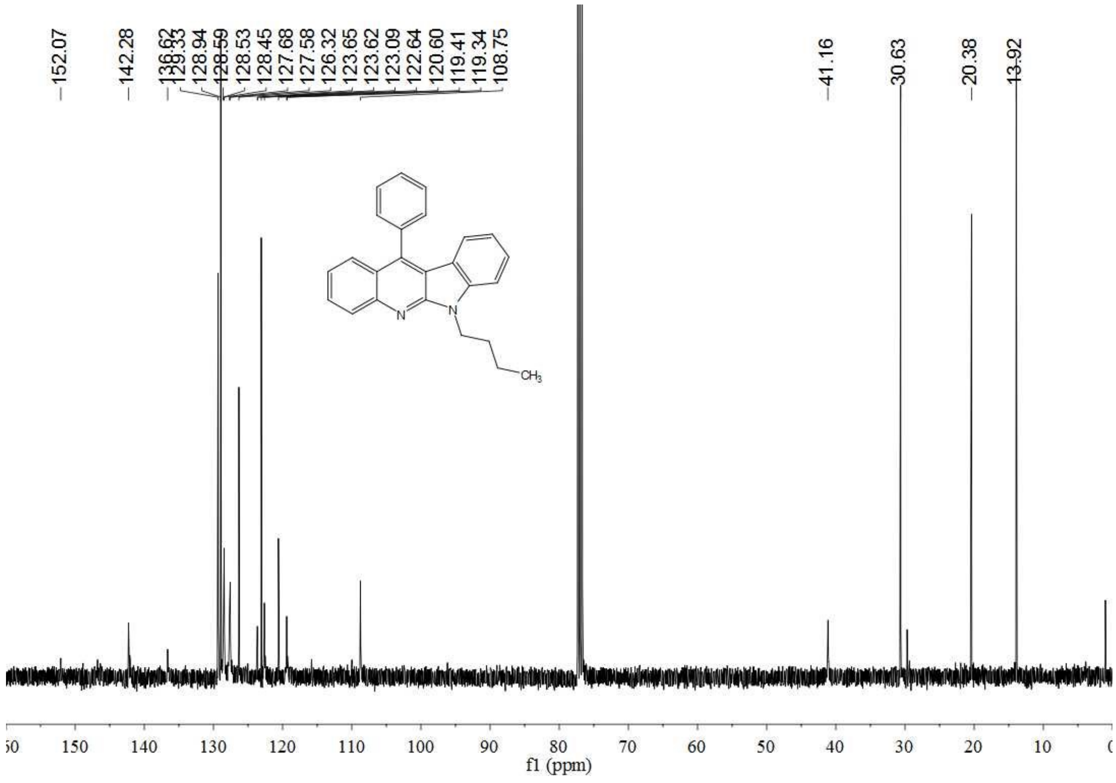

Fig. S6 ^{13}C NMR spectrum of **3c**

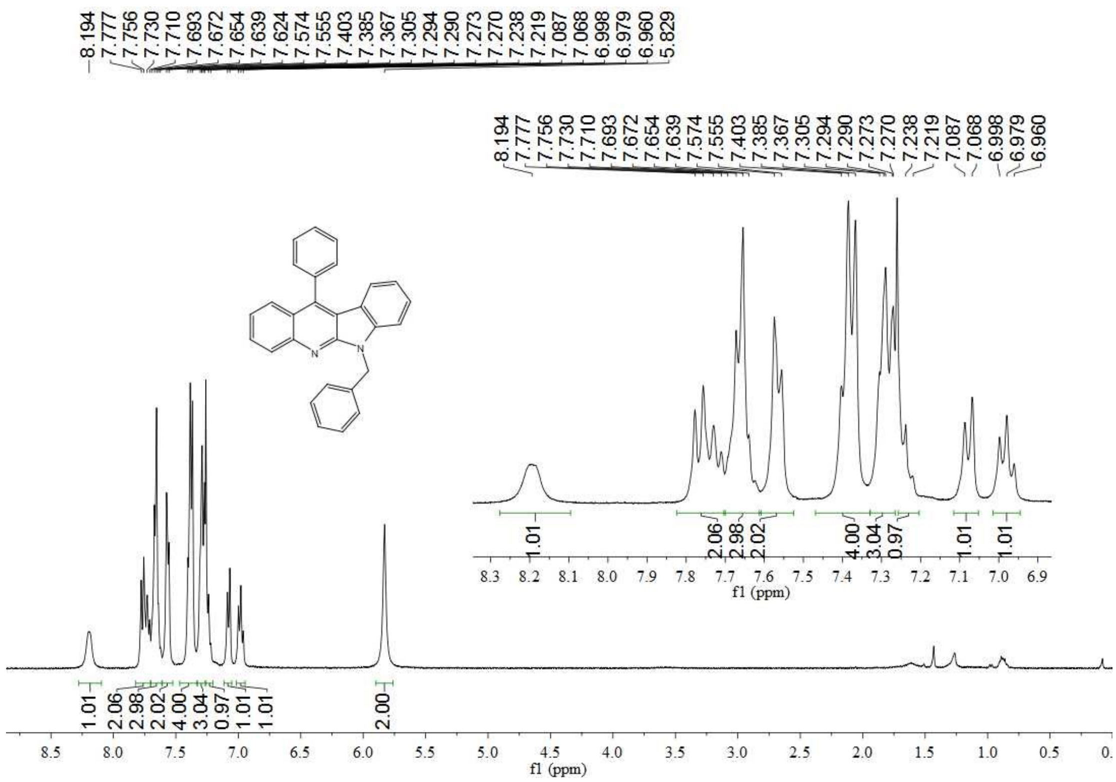

Fig. S7 ^{1}H NMR spectrum of **3d**

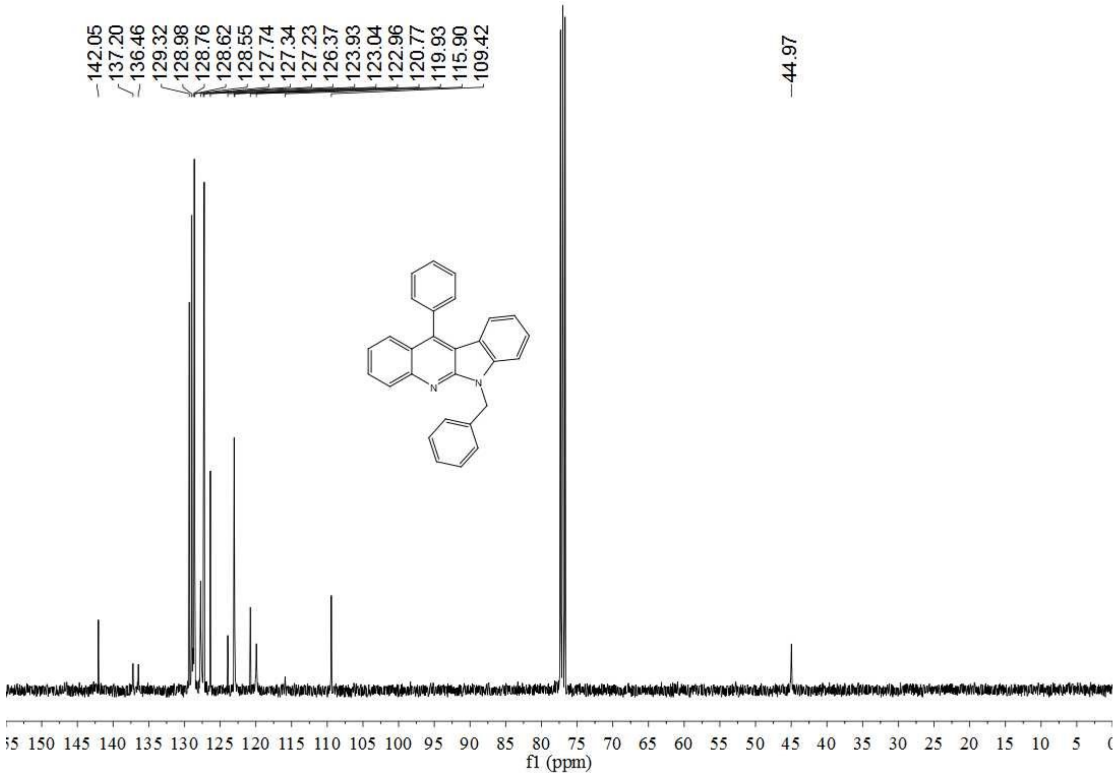

Fig. S8 ^{13}C NMR spectrum of **3d**

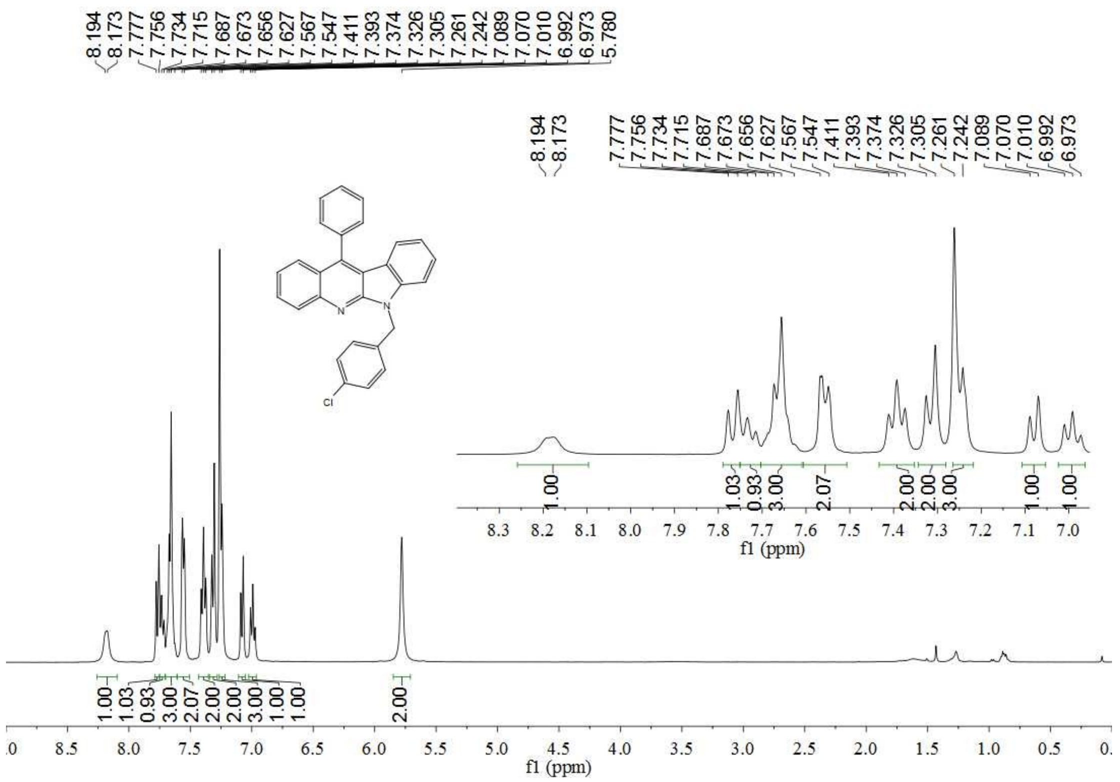

Fig. S9 ^{1}H NMR spectrum of **3e**

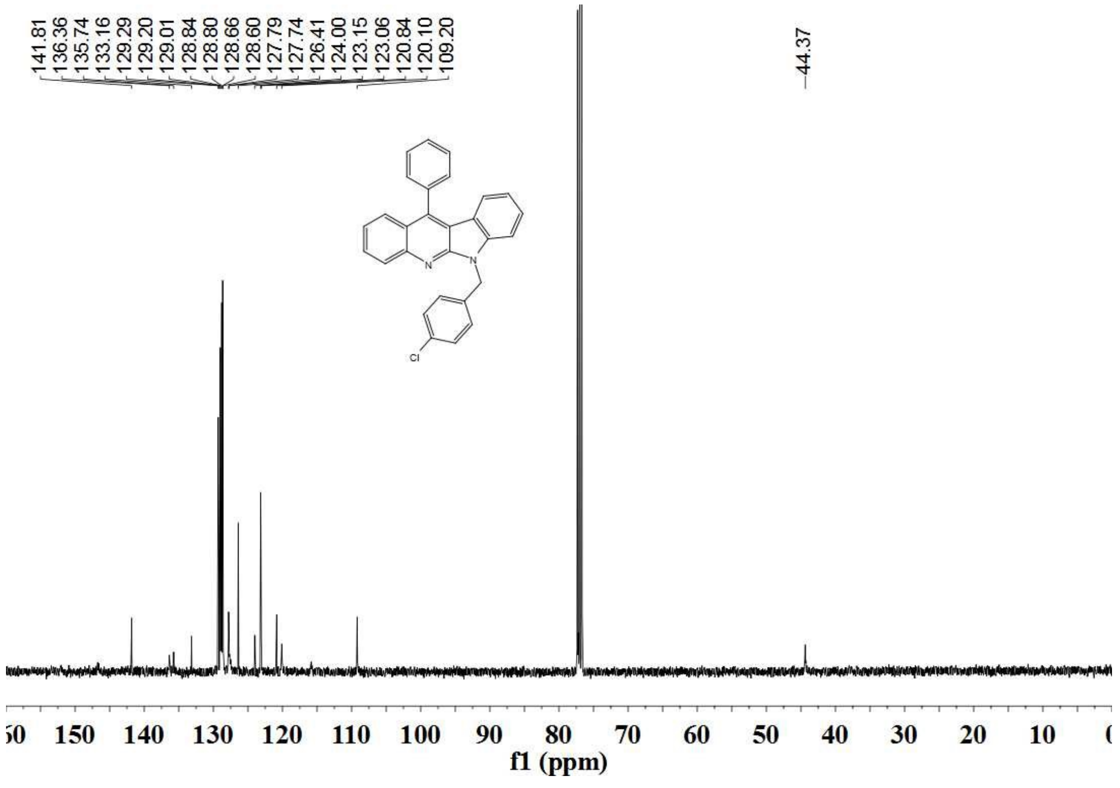

Fig. S10 ^{13}C NMR spectrum of **3e**

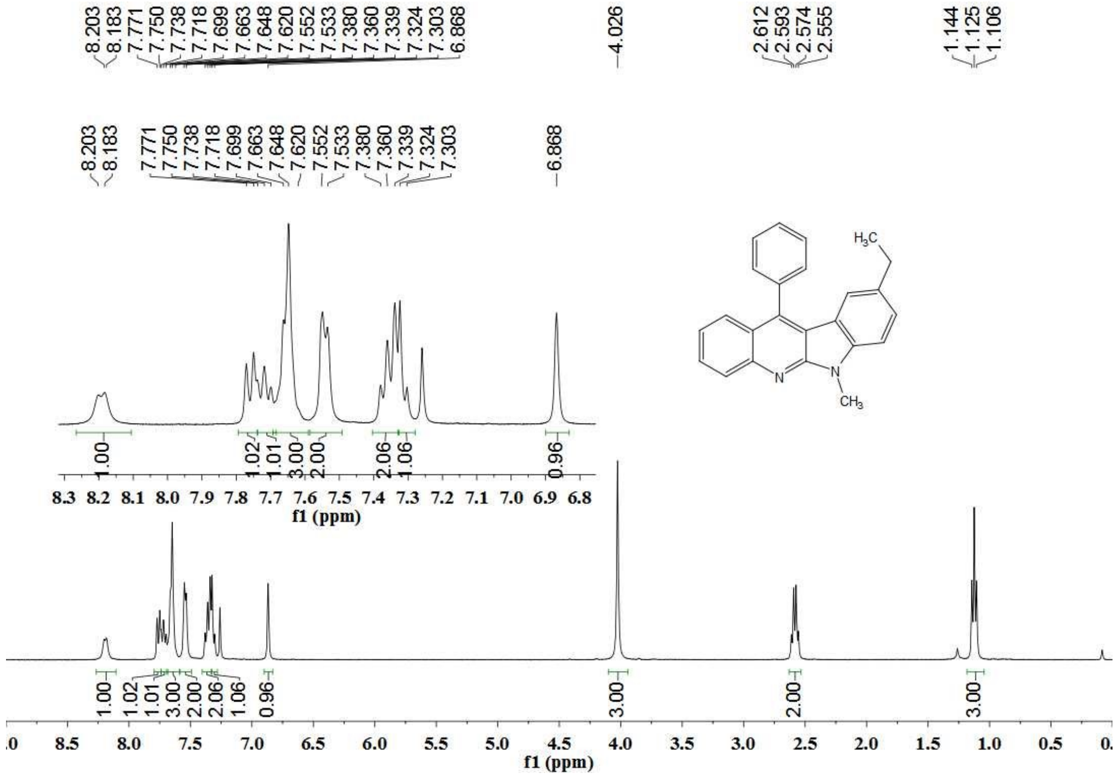

Fig. S11 ^{1}H NMR spectrum of **3f**

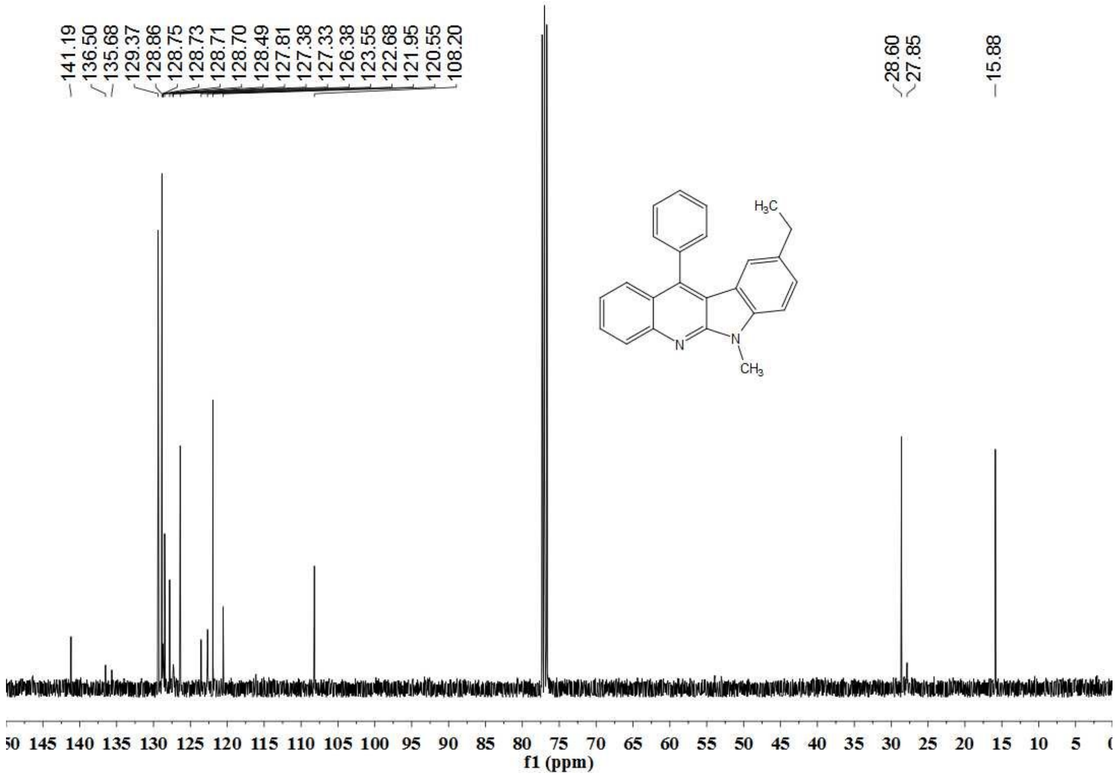

Fig. S12 ^{13}C NMR spectrum of **3f**

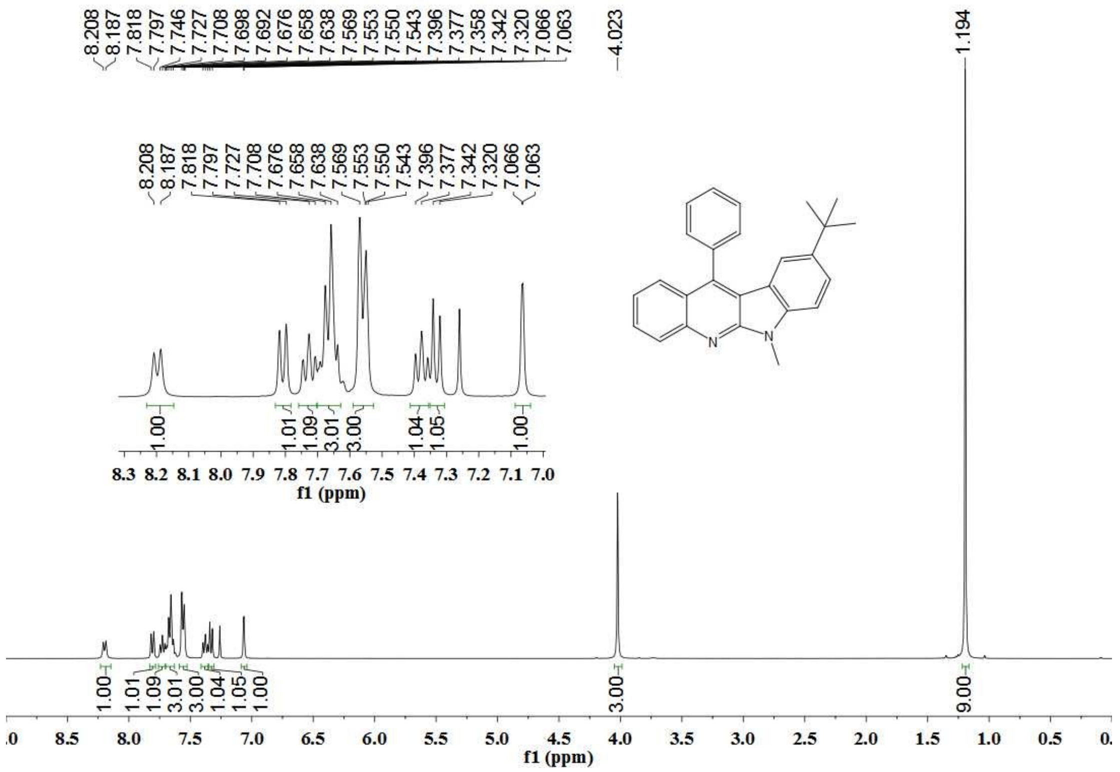

Fig. S13 ^{1}H NMR spectrum of **3g**

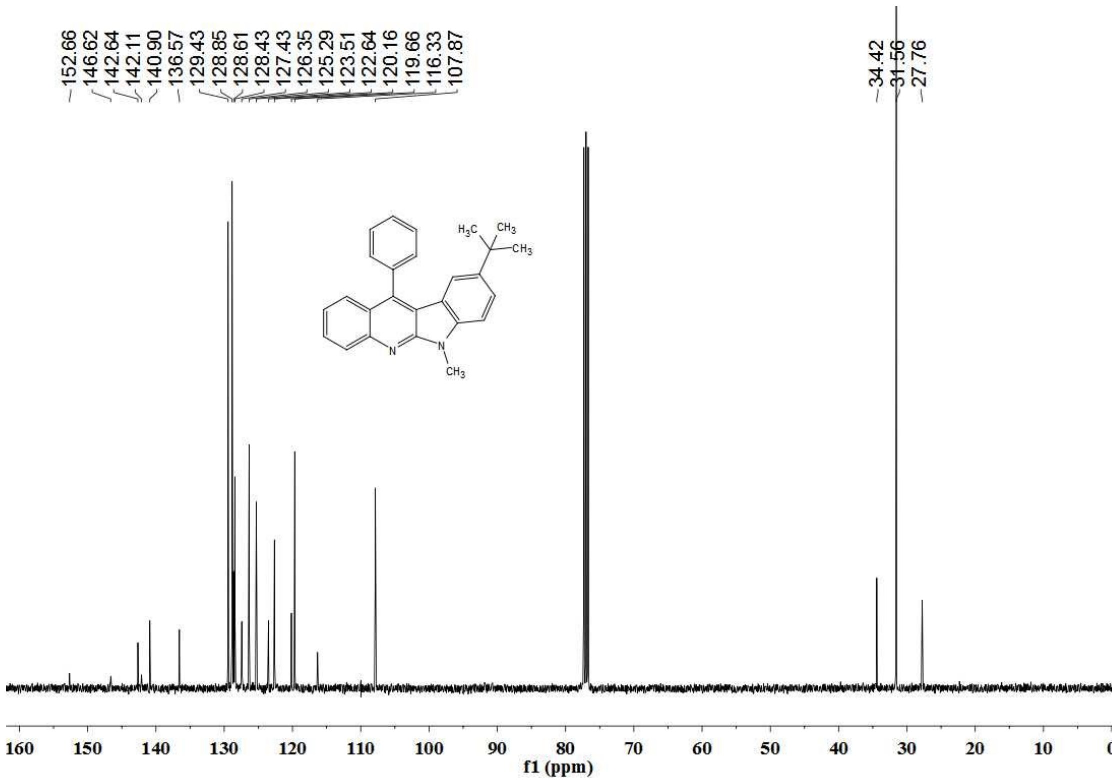

Fig. S14 ^{13}C NMR spectrum of **3g**

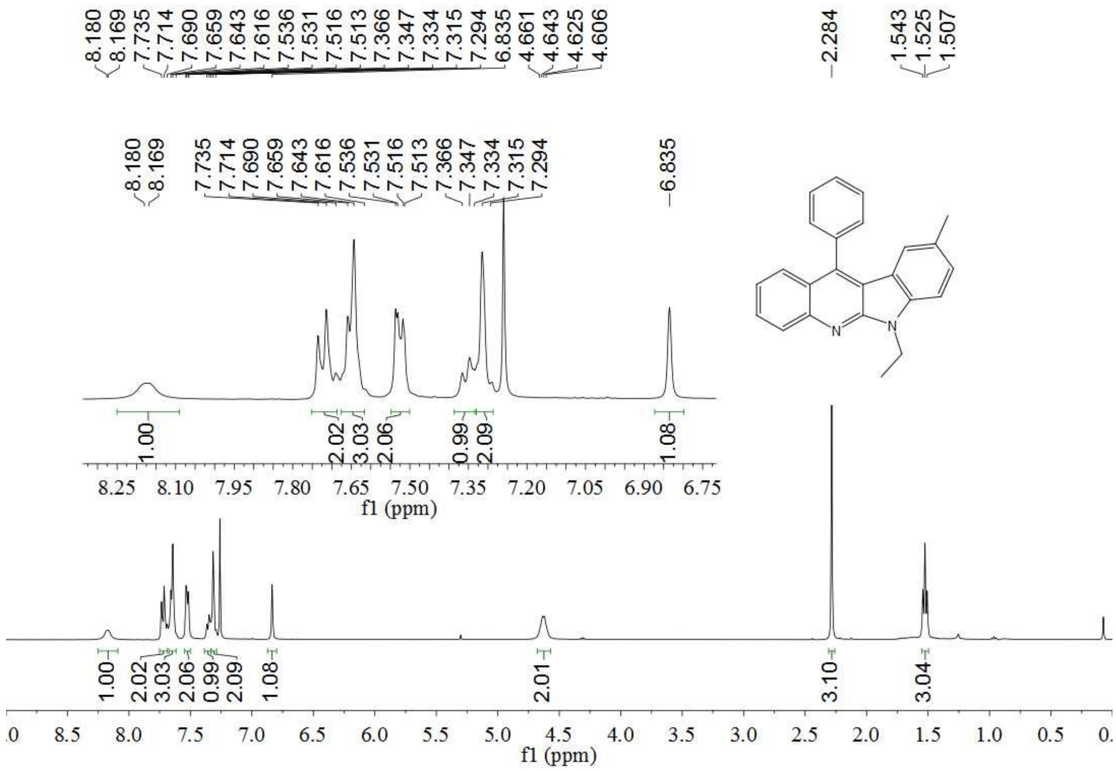

Fig. S15 ^{1}H NMR spectrum of **3h**

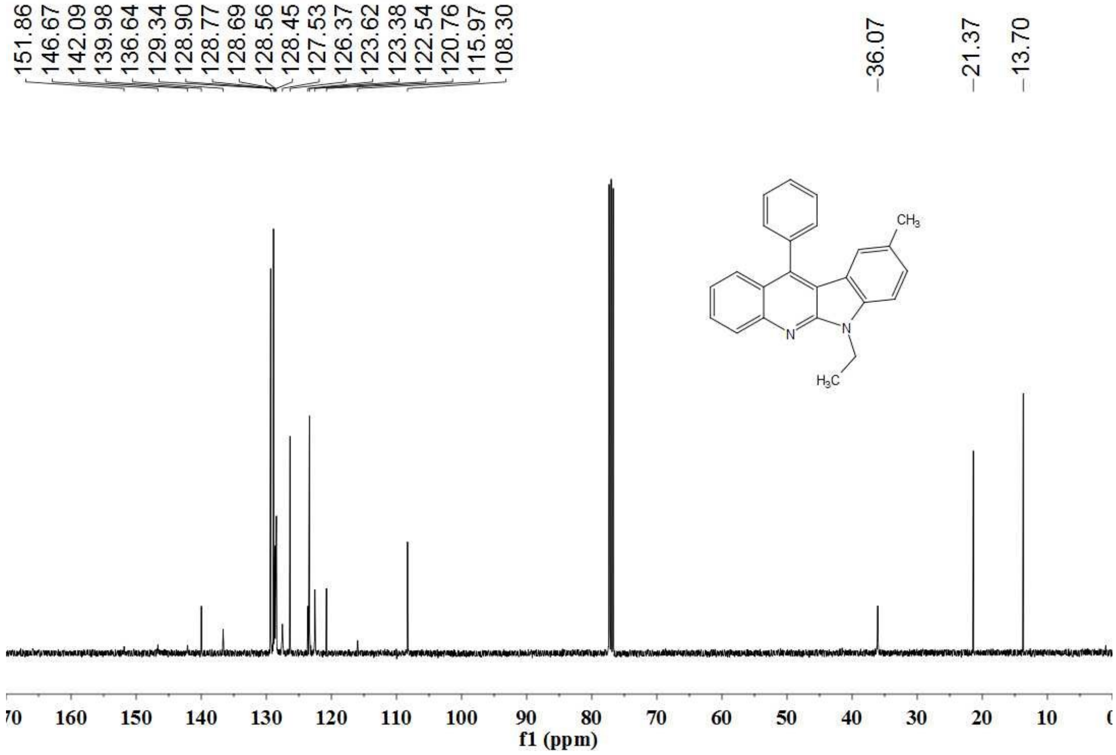

Fig. S16 ^{13}C NMR spectrum of **3h**

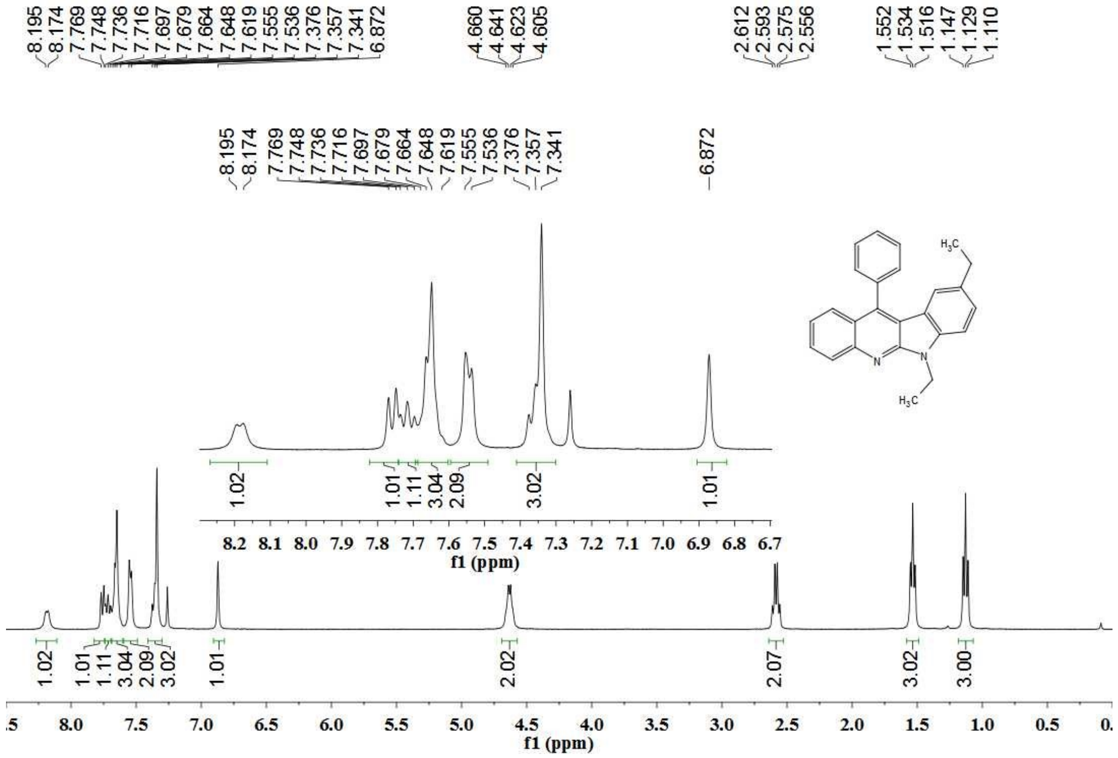

Fig. S17 ^{1}H NMR spectrum of **3i**

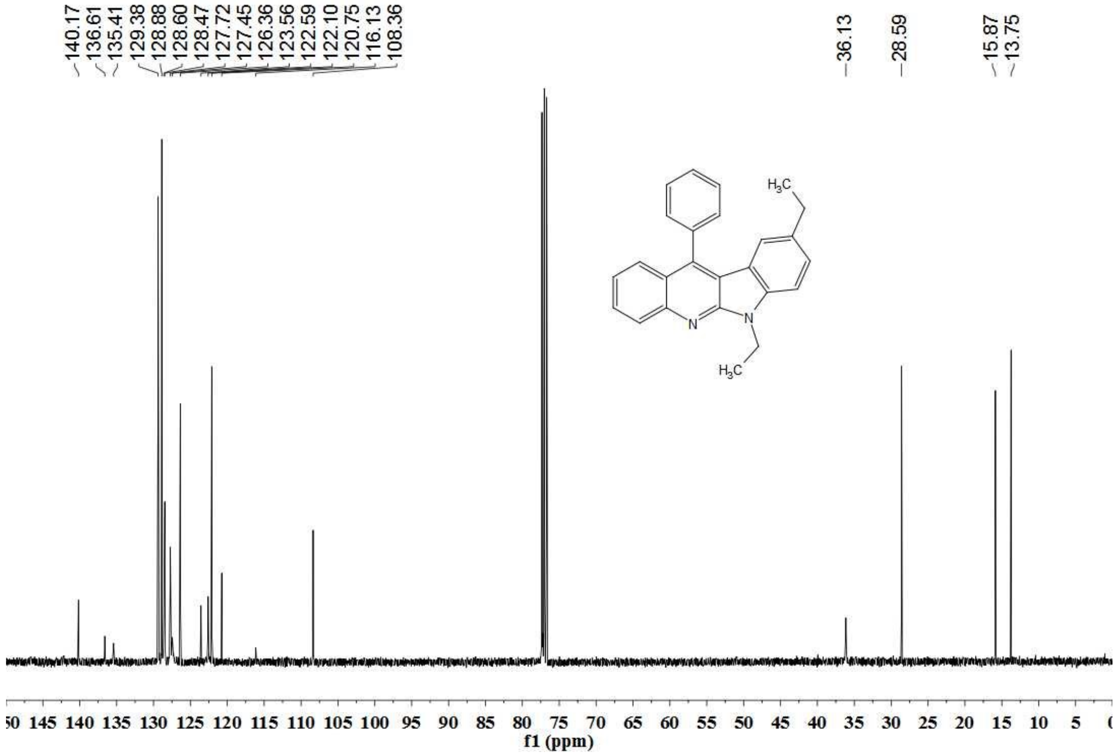

Fig. S18 ^{13}C NMR spectrum of **3i**

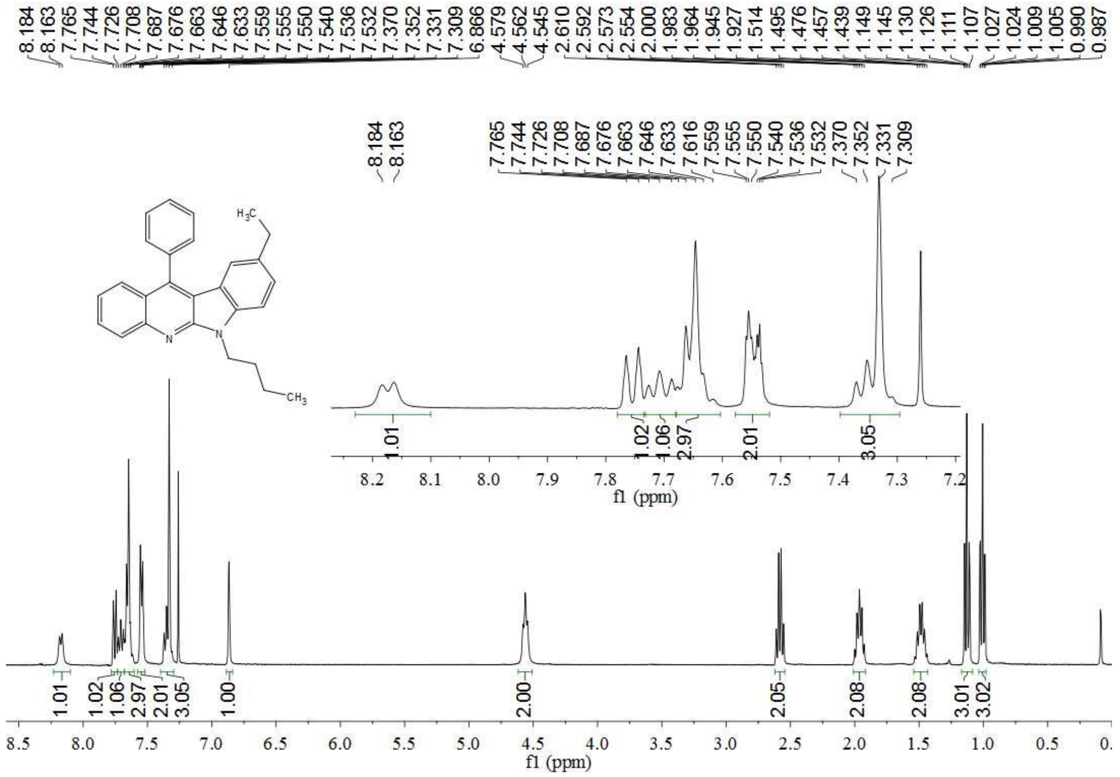

Fig. S19 ^{1}H NMR spectrum of **3j**

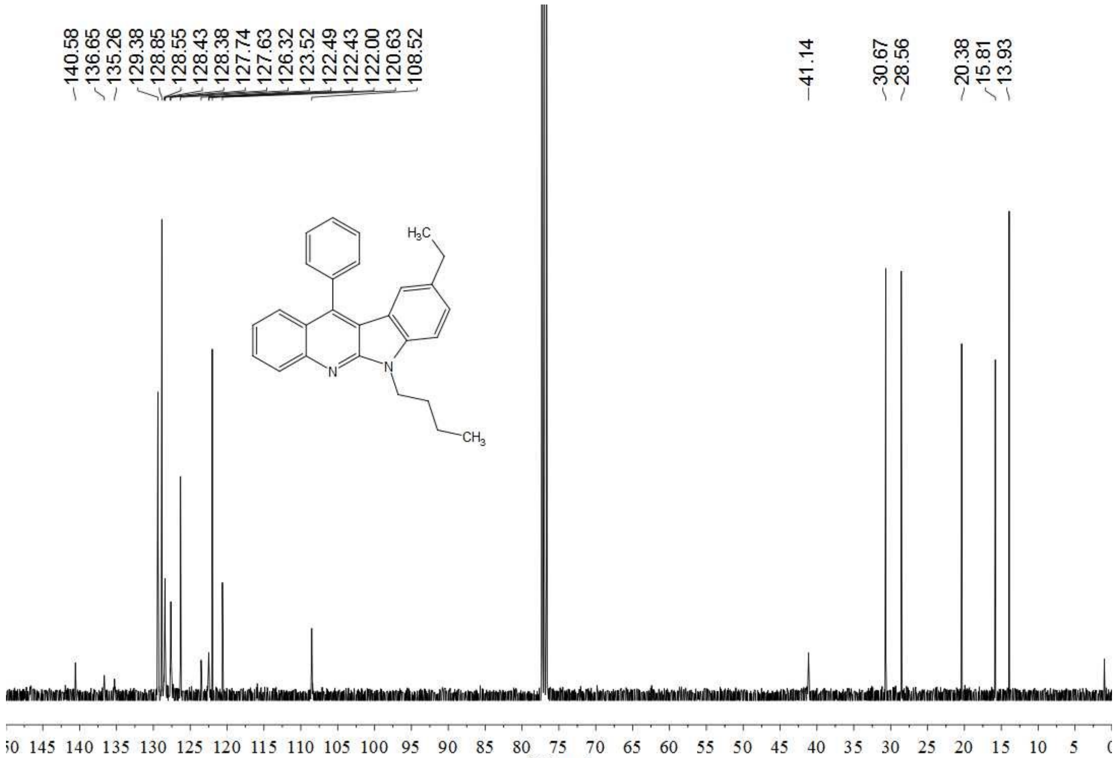

Fig. S20 ^{13}C NMR spectrum of **3j**

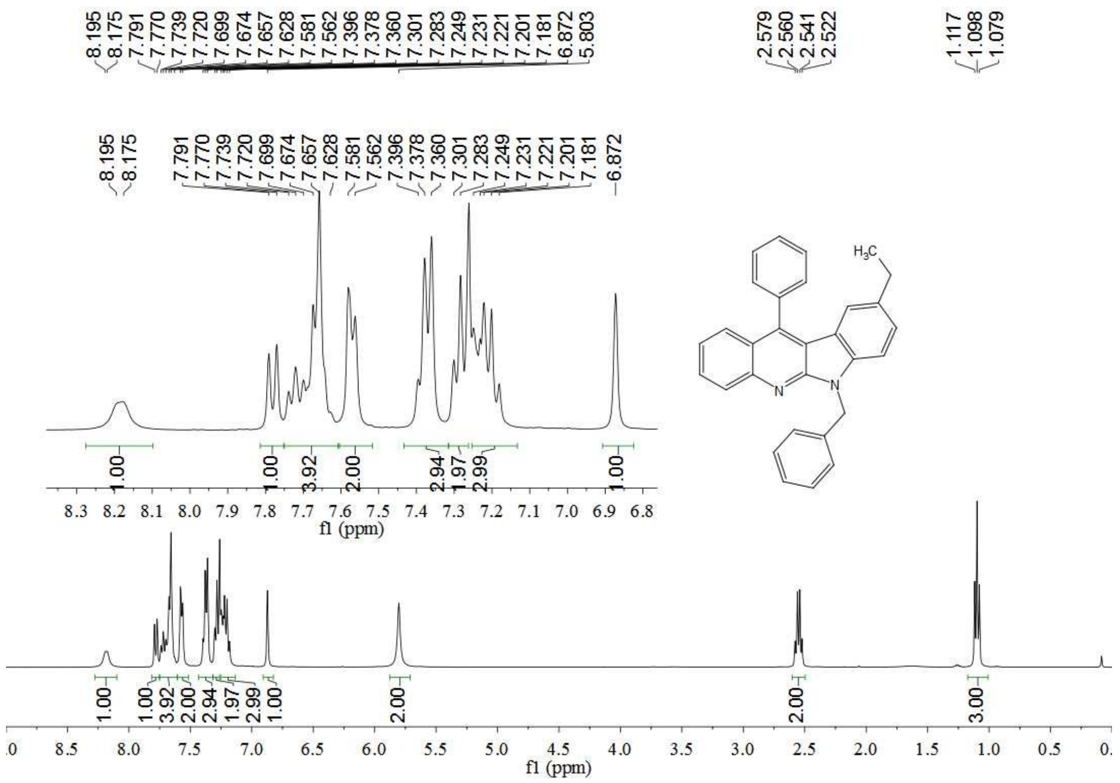

Fig. S21 ^{1}H NMR spectrum of **3k**

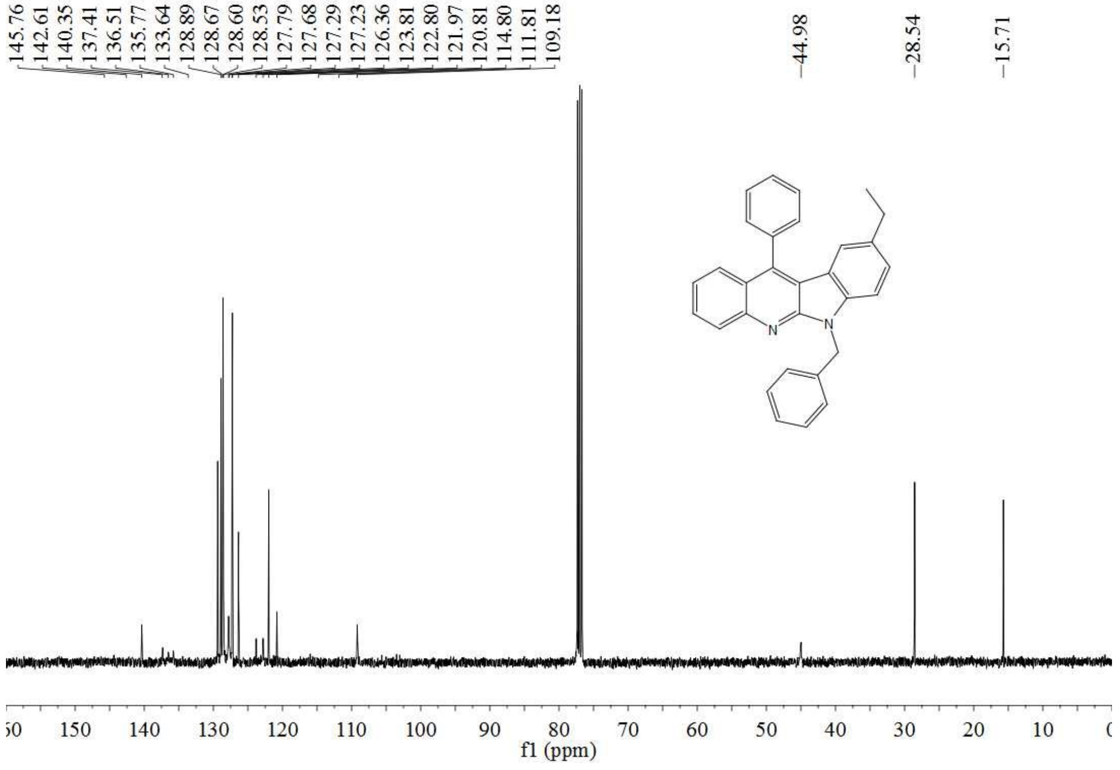

Fig. S22 ^{13}C NMR spectrum of **3k**

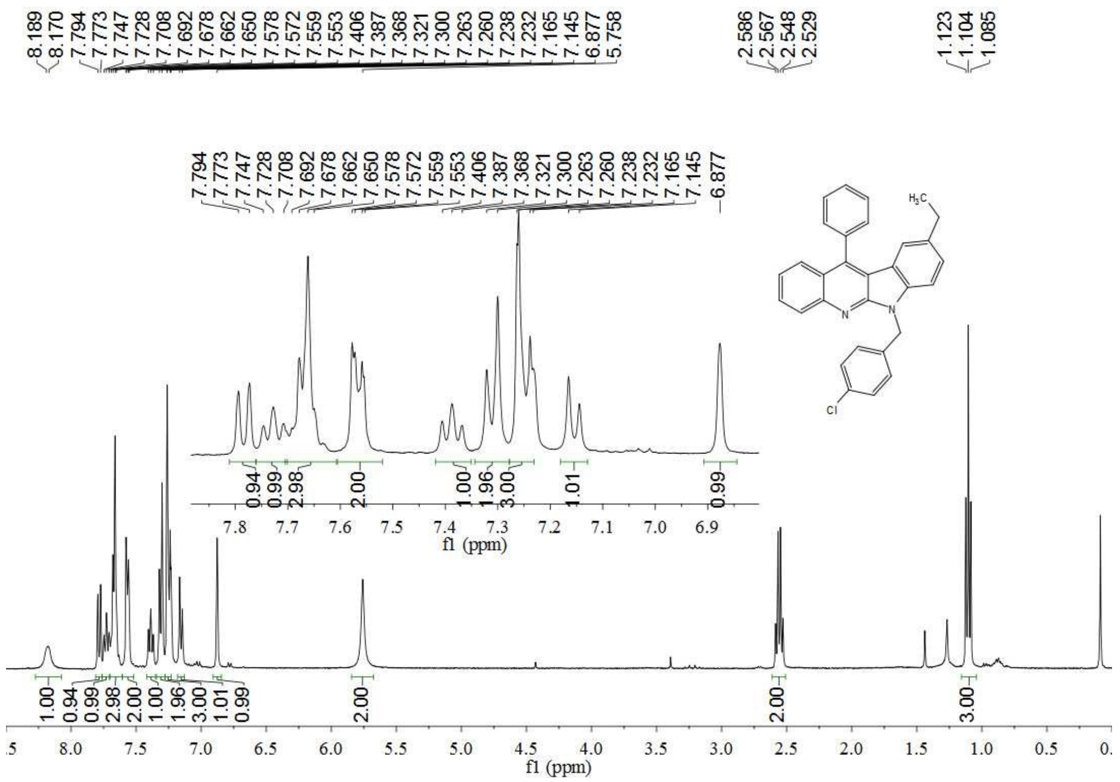

Fig. S23 ^{1}H NMR spectrum of **3l**

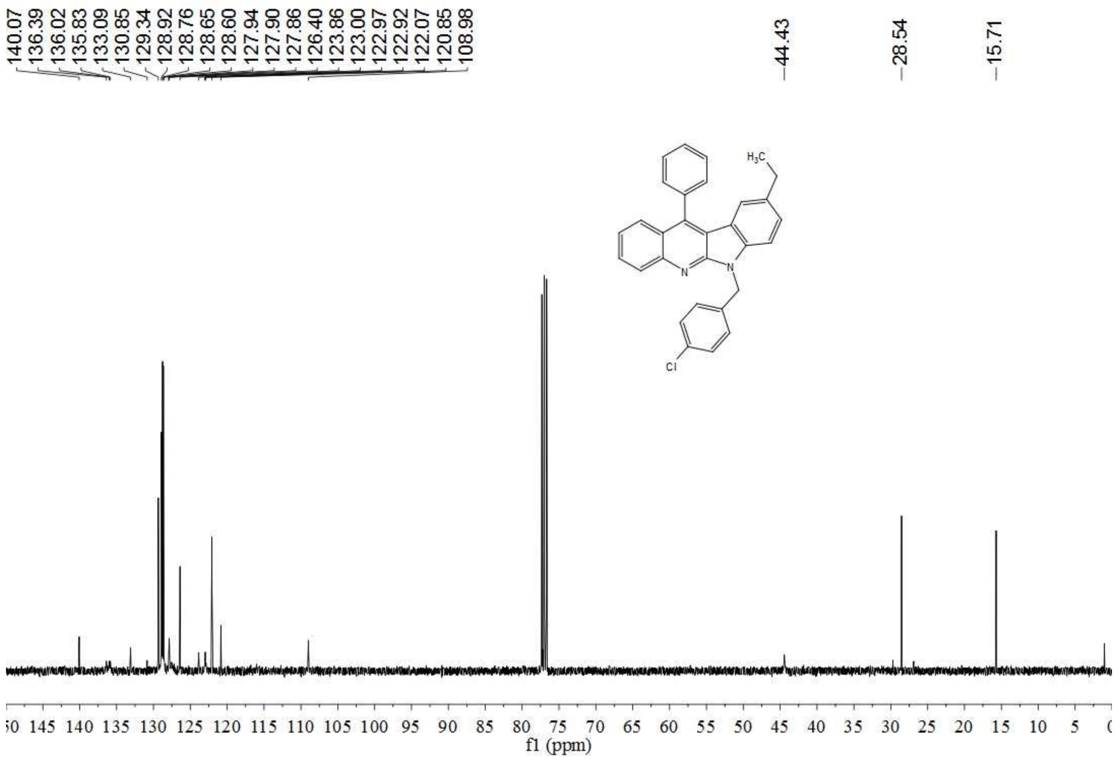

Fig. S24 ^{13}C NMR spectrum of **3l**

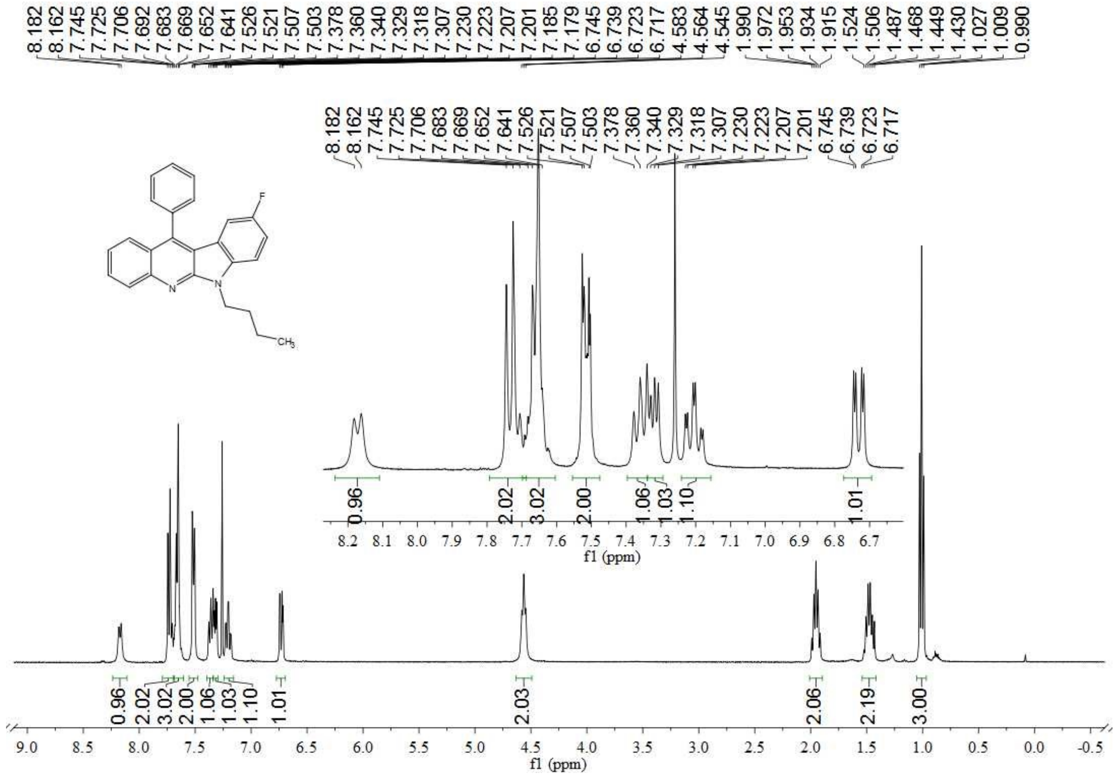

Fig. S25 ^{1}H NMR spectrum of **3m**

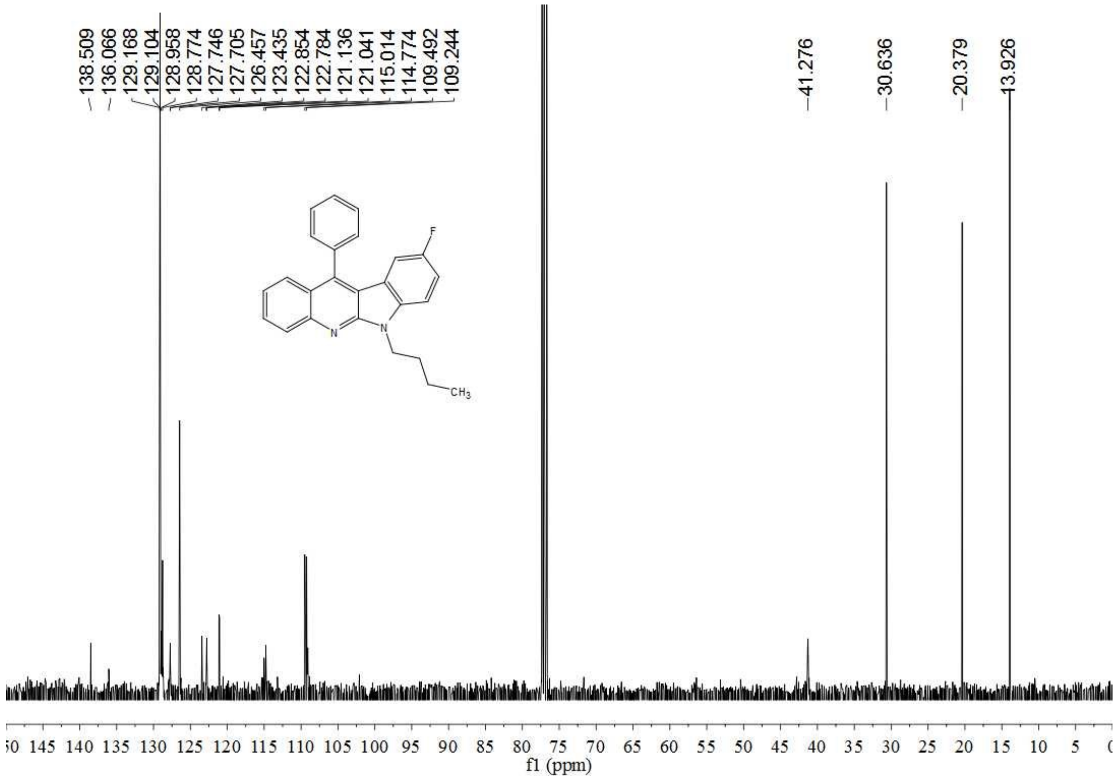

Fig. S26 ^{13}C NMR spectrum of **3m**

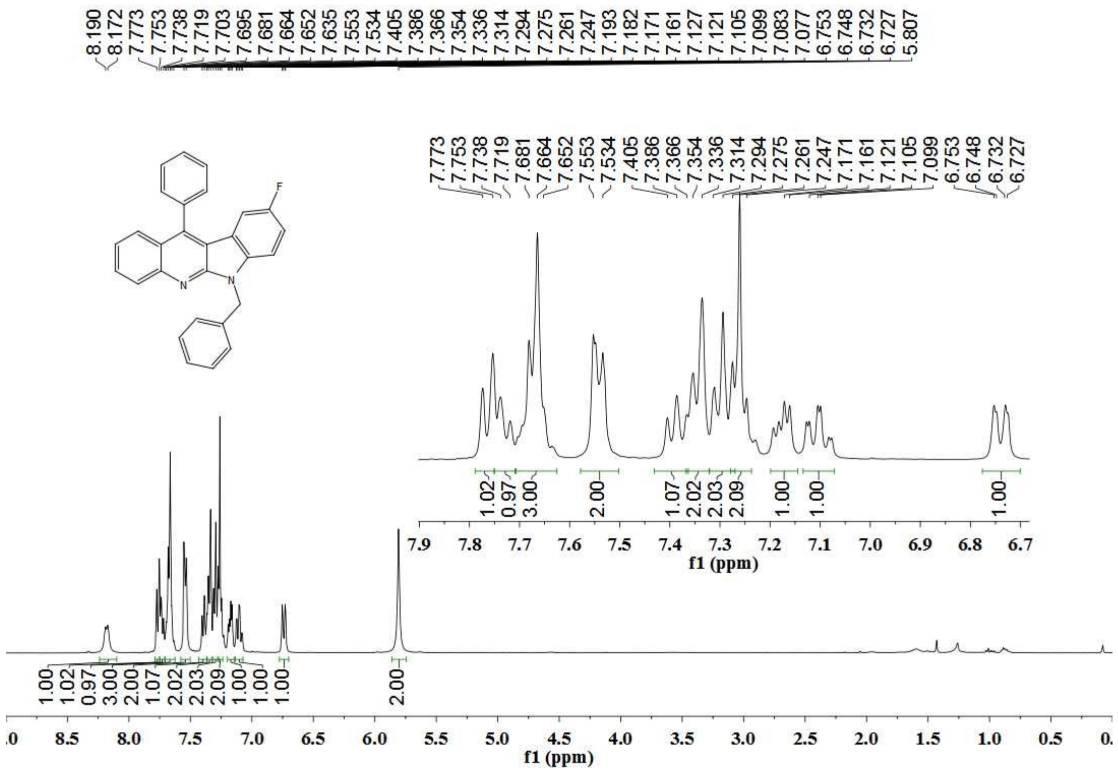

Fig. S27 ^{1}H NMR spectrum of **3n**

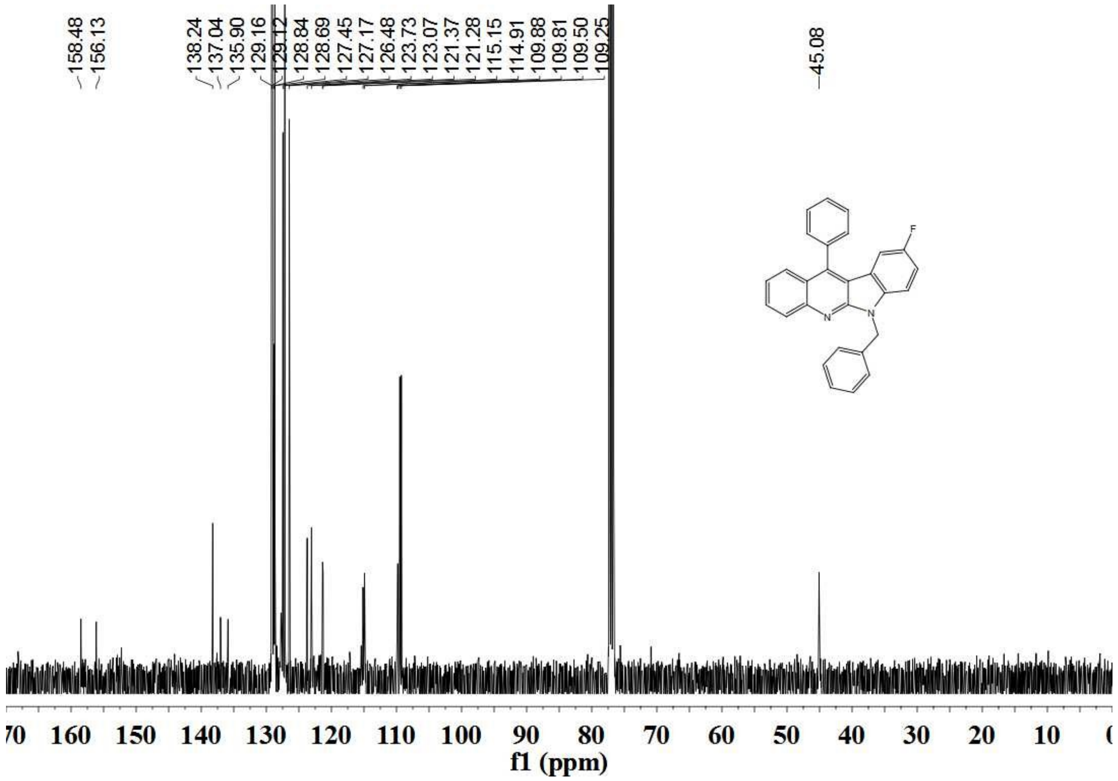

Fig. S28 ^{13}C NMR spectrum of **3n**

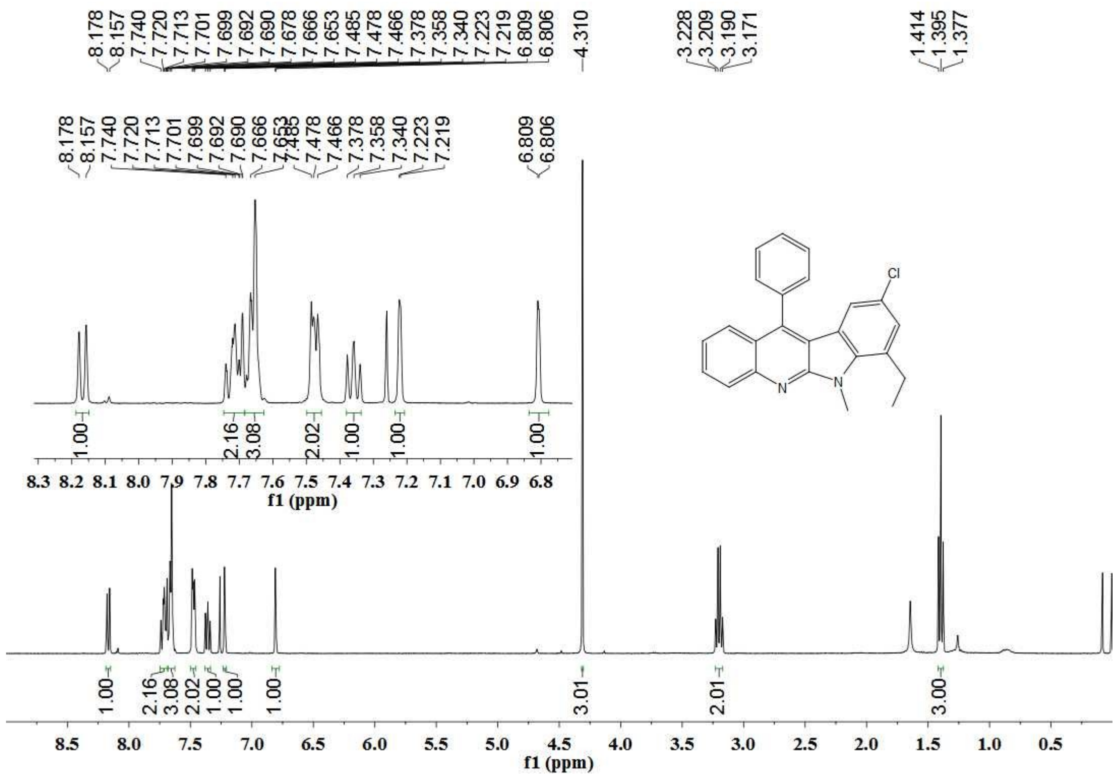

Fig. S29 ^{1}H NMR spectrum of **3o**

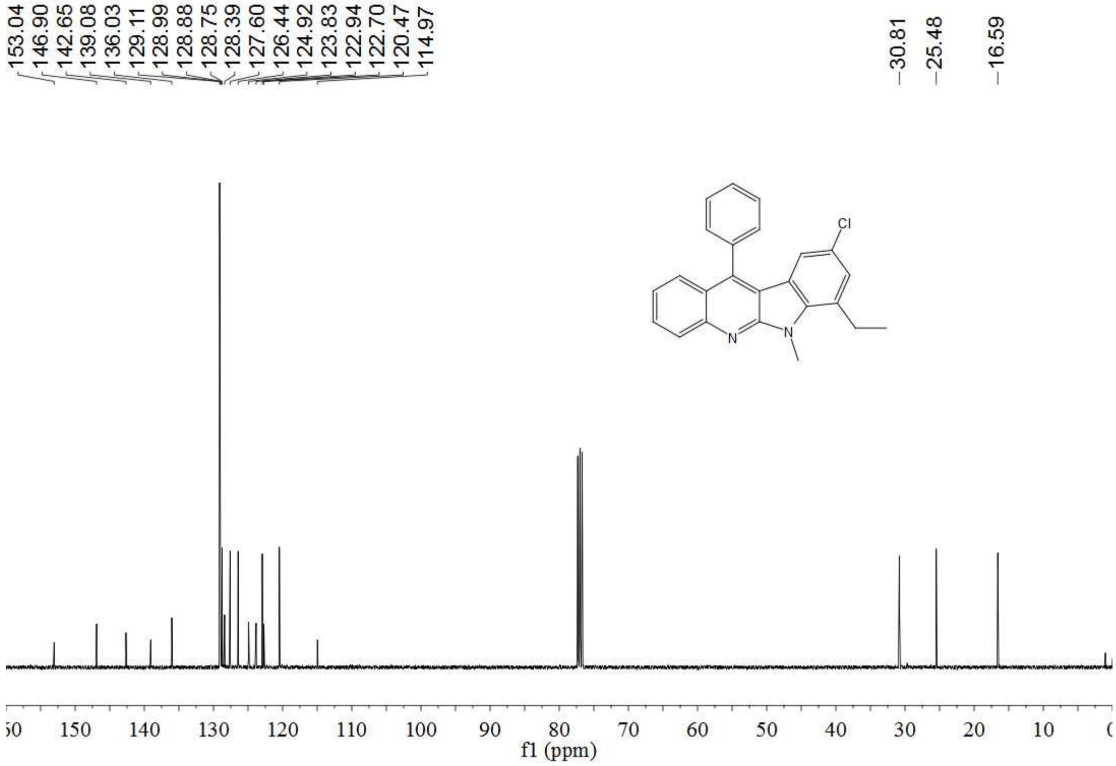

Fig. S30 ^{13}C NMR spectrum of **3o**

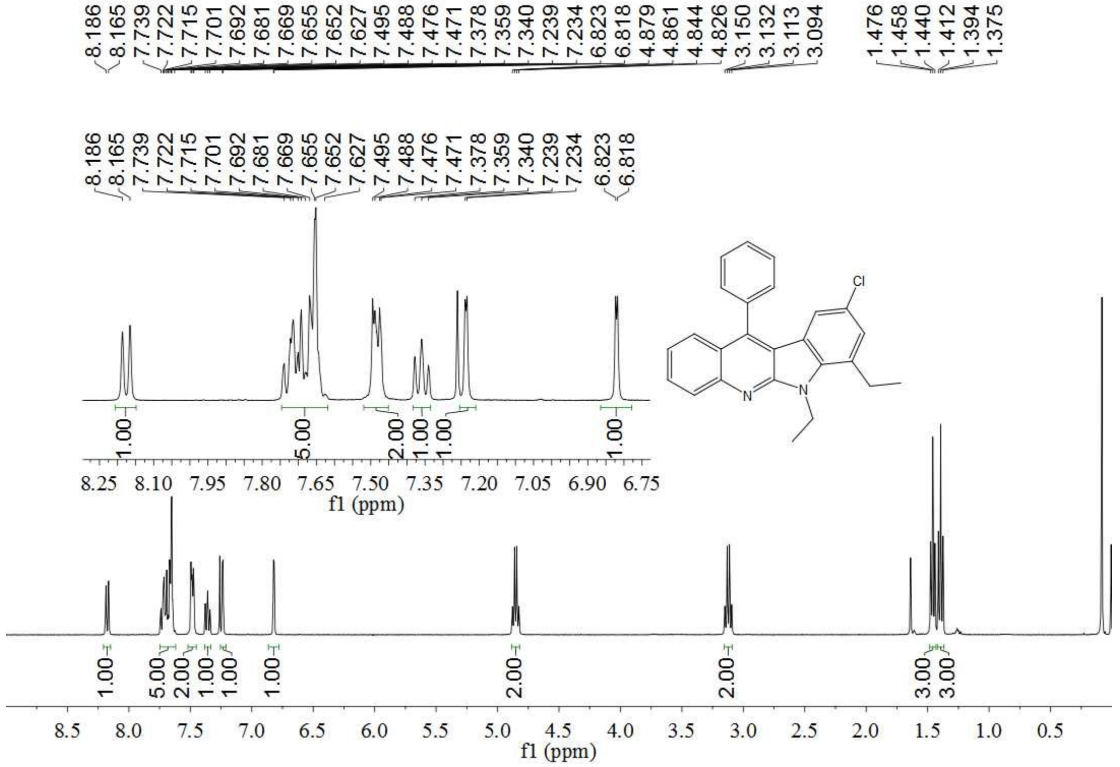

Fig. S31 ^{1}H NMR spectrum of **3p**

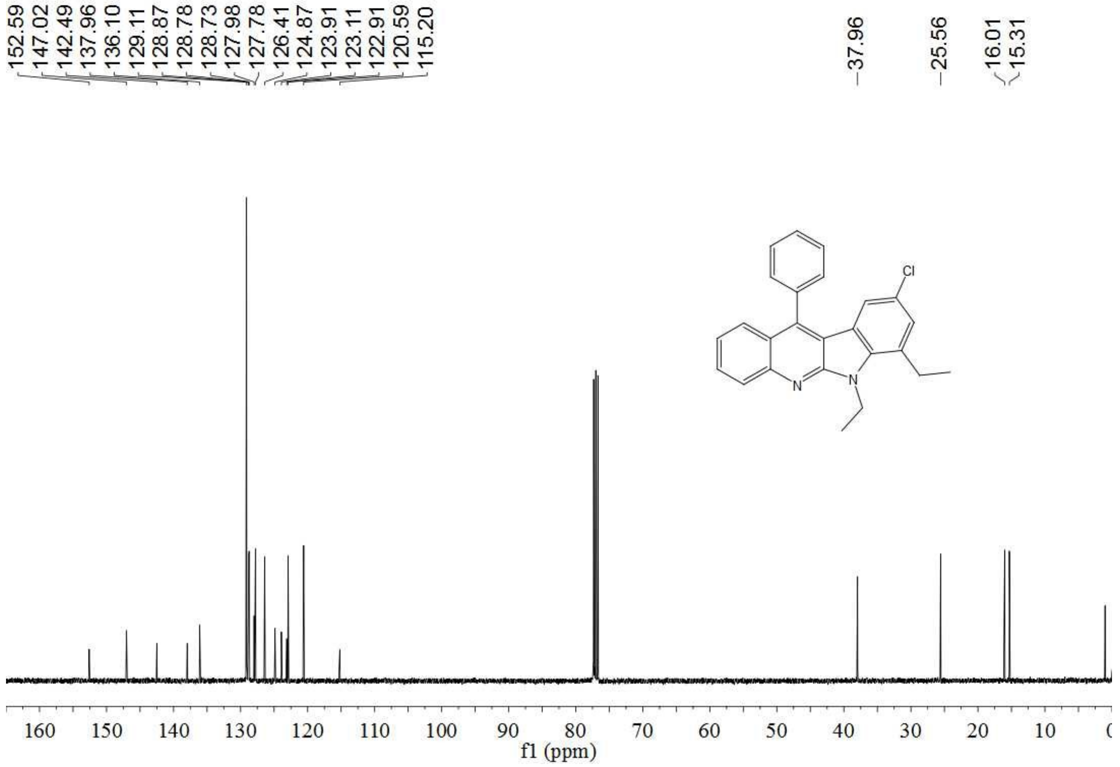

Fig. S32 ^{13}C NMR spectrum of **3p**

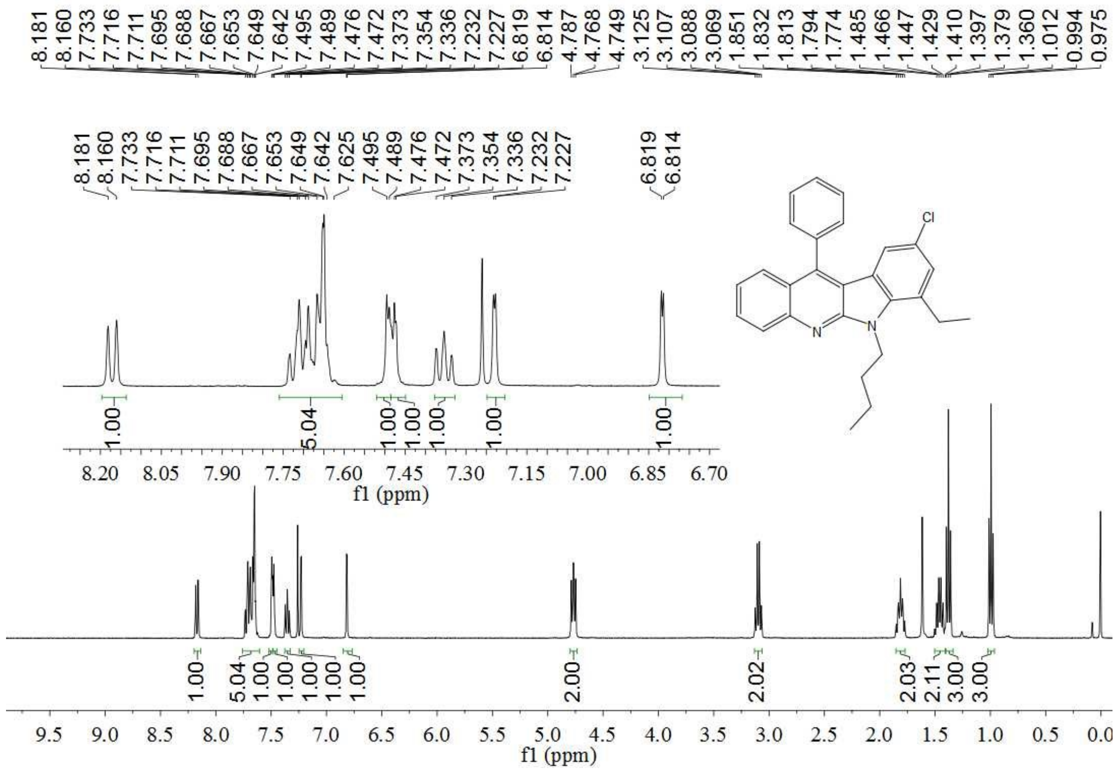

Fig. S33 ^{1}H NMR spectrum of **3q**

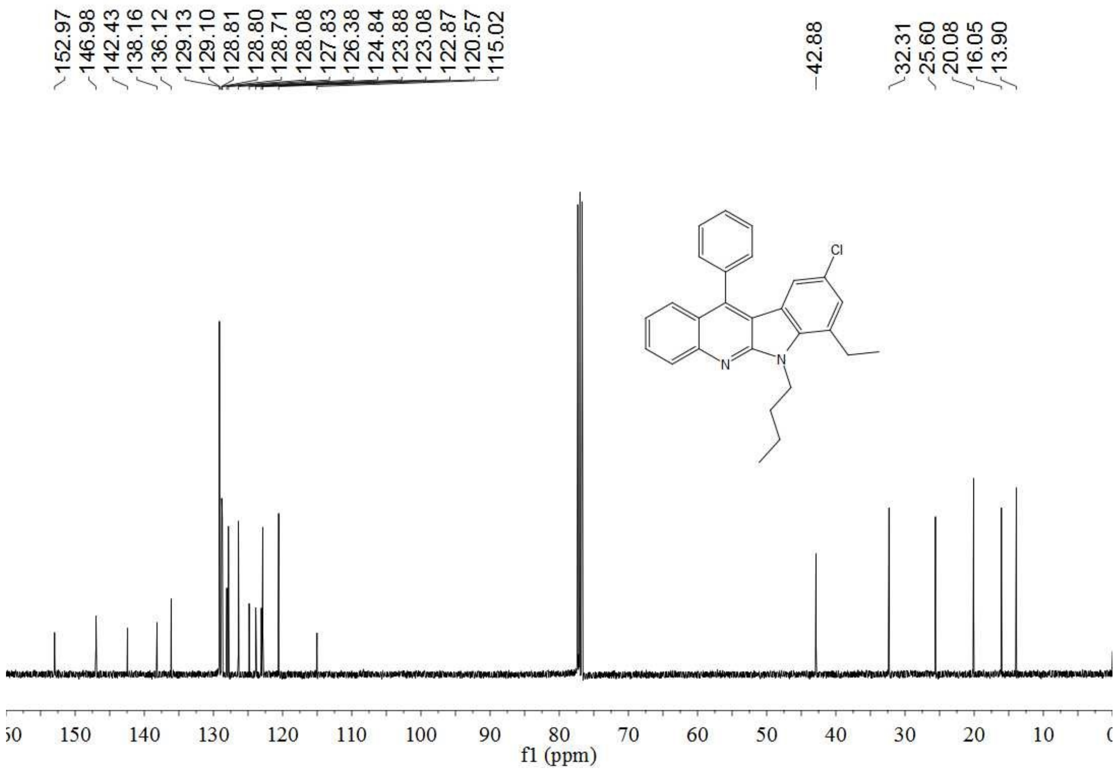

Fig. S34 ^{13}C NMR spectrum of **3q**

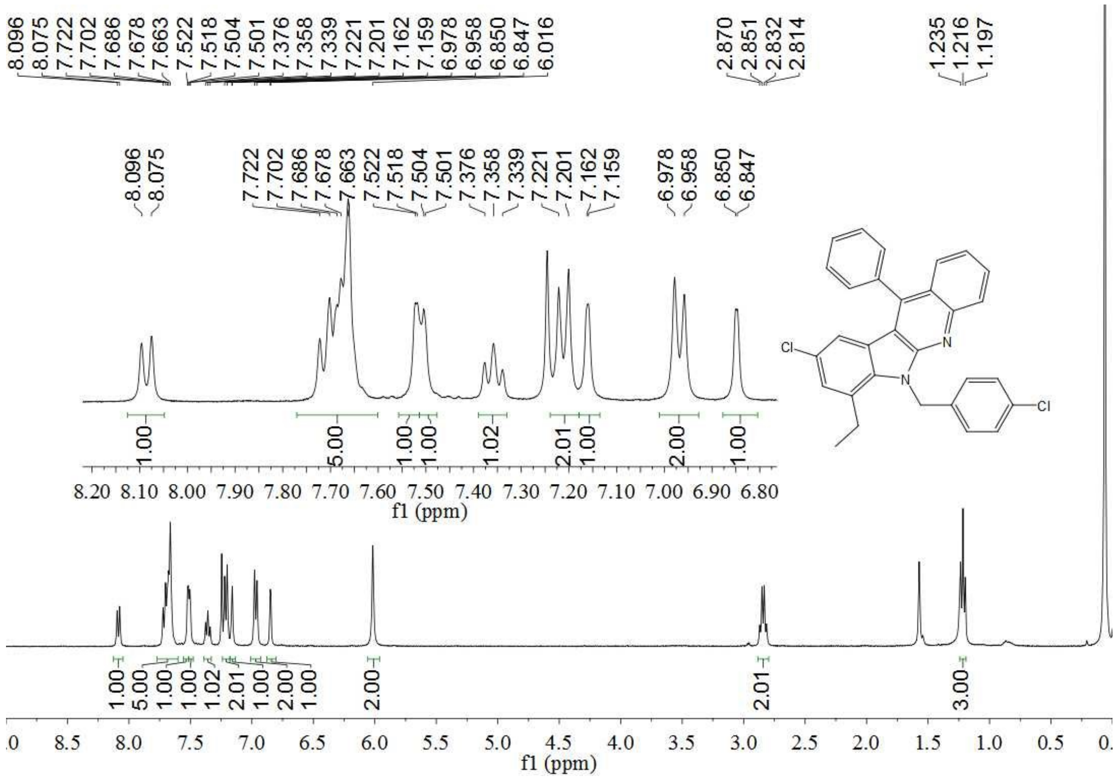

Fig. S35 ^{1}H NMR spectrum of **3r**

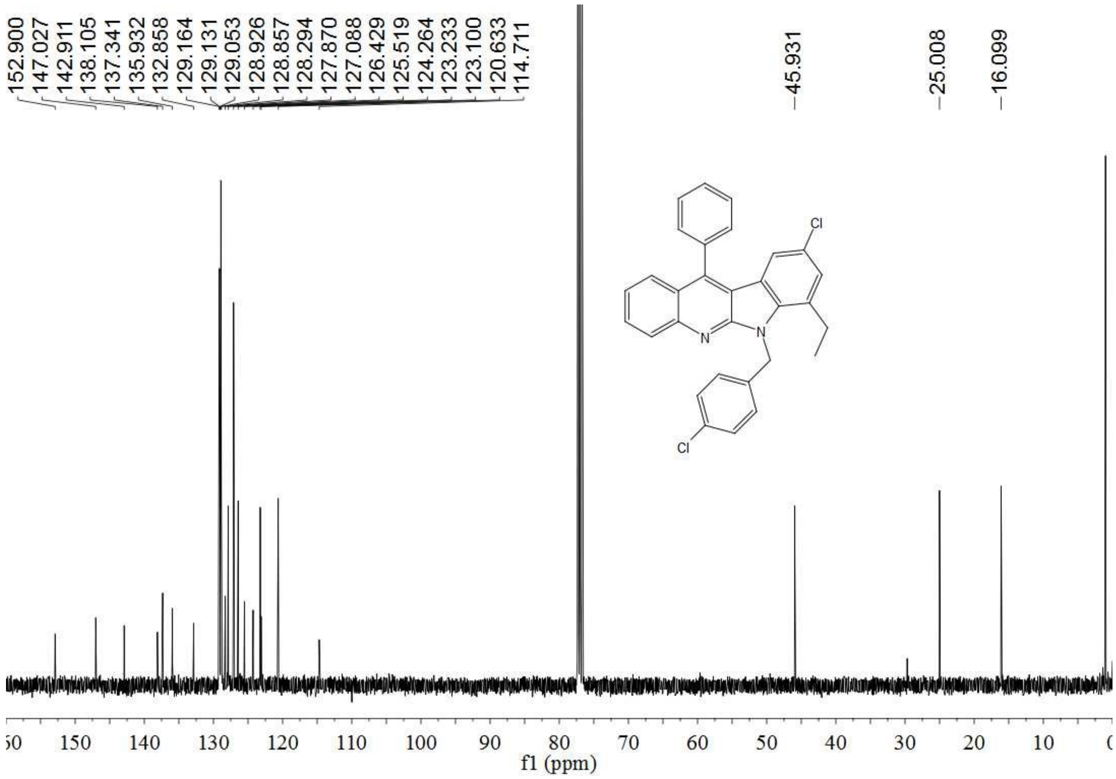

Fig. S36 ^{13}C NMR spectrum of **3r**
